# Supplementary material for: Asymmetric water transport in dense leaf cuticles and cuticle-inspired compositionally graded membranes
Source: Nat Commun. 2021 Feb 24;12:1267. doi: 10.1038/s41467-021-21500-0 (PMC7904774; doi:10.1038/s41467-021-21500-0)
Supplement: Supplementary file 1 — Supplementary Information [file 41467_2021_21500_MOESM1_ESM.pdf]

Supplementary Information

Asymmetric water transport in dense leaf cuticles and cuticle-inspired compositionally graded membranes

Aristotelis Kamtsikakis,[1] Johanna Baales,[2] Viktoria V. Zeisler-Diehl,[2] Dimitri Vanhecke,[1] Justin O. Zoppe,[1] Lukas Schreiber,[2*] Christoph Weder[1*]

[1] Adolphe Merkle Institute, University of Fribourg, Chemin des Verdiers 4, CH-1700 Fribourg, Switzerland

[2] Institute of Cellular and Molecular Botany, Department of Ecophysiology, University of Bonn, Kirschallee 1, D-53115, Bonn, Germany

Supplementary Note 1. Scanning electron microscopy (SEM), focused ion beam SEM (FIB-SEM) and transmission electron microscopy (TEM)

Cross-sections of the artificial membranes were isolated by imbibing the samples in ethanol-filled syringes and subsequent cryo-fracturing of the frozen syringe in liquid nitrogen using a pincer. The resulting materials were dried under a nitrogen stream, adhered to carbon tape with the top side of the casted membranes facing the tape and sputtered (Cressington 208HR High Resolution Sputter Coater, U.K.) with a 4 nm gold layer. The coated-membranes were observed in secondary electron mode with a Tescan Mira3 LM FE scanning electron microscope at 7 kV.

Cuticular membranes were prepared for FIB-SEM by staining (30 min) with a solution of 2 wt% OsO_4 and 1.5 wt% $K_4[Fe(CN)_6]$in DI, followed by 1 wt% thiocarbohydrazide in DI and finally 2 wt% OsO_4 (30 min)[1]. After drying, the cuticular membranes were processed as described for SEM above. FIB-SEM sectioning was performed on a SCIOS2 focused ion beam SEM (ThermoFischer) upon deposition of a protective platinum (Pt) layer at the inner cuticular side using a 30 kV Gallium ion beam. Subsequent imaging of the cross-sections at an angle of 52° was conducted using the T1 backscattered detector with a 5kV electron beam. For TEM, the cuticular membranes were prepared following the same protocol as outlined for the FIB-SEM staining[1], which was followed by a dehydration series in ethanol and subsequent embedding in Epon (PlanoEM, Germany). The Epon blocks were polymerized at 60 °C before being trimmed and cut. Ultrathin sections were created using a Leica Ultracut UC6 ultramicrotome (Leica, Austria) at 'gray-silver' thickness (~60-80 nm thick). The sections were collected on standard copper single slot TEM grids (PlanoEM, Germany) and poststained with lead citrate in a Leica EMStain (Leica, Austria). The sections were observed in a FEI Tecnai Spirit transmission electron microscope (ThermoFischer, USA), at a voltage of 120 kV, equipped with a 2K Olympus Veleta digital camera (Olympus, Tokyo). Images were recorded using the Tecnai and Imaging (TIA) software (version 4.7 SP3).

To image the CNCs with TEM, freeze-dried CNCs were redispersed in ultra pure water at a concentration of 0.03 wt% and spin-coated (4 µL, 3000 rpm, 1 min) onto plasma-treated formvar/carbon TEM grids. The CNCs were visualized without staining using a FEI Tecnai Spirit transmission electron microscope at a voltage of 120 kV. Size analysis of the CNCs was conducted by using the image processing software ImageJ 1.51k. Five different images with a total of 120 CNCs were analyzed and the corresponding values of length, width, and aspect ratio were plotted as relative frequency histograms. The particle dimensions were determined to be 128 ± 55 nm × 14 ± 4 nm × 9 ± 3 (length × width × aspect ratio) (Supplementary Figure 2a-d). The determined width may be an overestimation due to lateral aggregation of CNCs, as previously reported[2].

Supplementary Note 2. Water permeability measurements (ASTM E96)

Standard test methods for water vapor transmission of materials according to ASTM E96 (dry and inverted wet cup methods) were followed to measure the water permeability of SBS and SBS/CNC membranes[3]. Water impermeable aluminum cups that had been specially designed for the ASTM E96 method were purchased from Thwing-Albert (U.S.A.). The nanocomposite membranes were preconditioned at the respective RH until equilibrium was reached (< 2 h) and mounted on the cups containing $CaCl_2$ (dry cup method; assumption of 0% RH inside the cups, RH_R = 0%) or DI water (inverted wet cup method; RH_D = 100%) and the cups were sealed with neoprene gaskets (see also Supplementary Figure 8a-b). The cups were placed in a ventilated oven whose temperature was set to 25 °C. For dry cup method experiments, a steady relative humidity (RH) environment was established by placing glass containers of saturated aqueous solutions of NaCl (~75% RH_D) or KCl (~85% RH_D) in the oven[4]. For the inverted wet cup method experiments (RH_D = 100%), an excess of $CaCl_2$ was utilized to maintain dry conditions in the oven (assumption of RH_R = 0%). The cups were removed in regular time intervals (typically $1 < \Delta t < 48$ h) and weighted to determine the water transmittance (J) through the membrane gravimetrically from the slope of the change of the total mass of the cups as a function of time. At least six data points acquired over the course of at least 24 hours were utilized for each linear regression (coefficients of determination of the regression lines (R^2) were always better than 0.99) (for

representative curves see Supplementary Figure 8a-b). The saturated water vapor pressure (p^{sat}) was calculated using the Antoine Equation (1):

$$\log_{10}(p^{\text{sat}}) = A - \frac{B}{T + C} \tag{1}$$

by using values provided by NIST for the *A*, *B*, *C* constants (p^{sat} = 3162 Pa at 25 °C). The applied water vapor pressure gradient Δp was determined with Equation (2) using the relative humidity conditions at the donor and receiver sides (RH_D and RH_R) during the experiments:

$$\Delta p = (\text{RH}_\text{D} - \text{RH}_\text{R})\, p^{\text{sat}} \tag{2}$$

By using the water transmittance (J), the thickness (ℓ) and the available area (A) of the membranes for permeation, the water permeability, P (kg m m^{-2} s^{-1} Pa^{-1}) was calculated with Equation (3):

$$P = \frac{J}{A}\frac{\ell}{\Delta p} \tag{3}$$

Although the ASTM E96 protocol states that the calculation of permeability is optional,[3] permeability is the preferably reported size for dense membranes, since it corresponds to the intrinsic properties of the tested material, assuming that normalization with the membrane thickness is possible (i.e., homogeneous thickness and easy to determine without affecting the membrane structure). Corrections were applied according to the ASTM E96 protocol (atmospheric pressure buoyancy correction, still air and specimen surface correction and edge mask correction). Permeability measurements were conducted for both sides of the membranes and the reported values were the mean and standard deviation of n = 3 measurements. We consistently report water permeability values in SI units to avoid confusion with non-SI unit variations.

To quantify the asymmetric transport through the artificial membranes, we used the Permeability Asymmetry Factor (*PAF*) defined by Equation (4):

$$PAF = \frac{P_{\text{Bottom facing donor}}}{P_{\text{Top facing donor}}} \tag{4}$$

Where $P_{\text{Bottom facing donor}}$ and $P_{\text{Top facing donor}}$ correspond to the determined permeability P with the bottom (i.e., rich in CNCs) and the top side of the SBS/CNC membranes facing the donor, respectively.

For the tests with varying RH_D conditions and CNC-content, we used membranes with a thickness of ~33 μm. To investigate the impact of membrane thickness on water transport, we also conducted permeability tests with the inverted wet cup method (RH_D = 100%, RH_R = 0%) using neat SBS and SBS/CNC 15 wt% membranes with three different thicknesses (ℓ ~ 60, 90, 120 μm) at 25 °C.

Supplementary Note 3. Dynamic mechanical analysis (DMA) measurements

Dynamic mechanical analysis (DMA) measurements were conducted on a dynamic mechanical analyzer (TA Instruments, Model Q800) with a film-tension clamp in tensile mode with constant oscillation parameters (1 Hz frequency; 15 μm amplitude). Prior to testing, the artificial SBS/CNC membranes were preconditioned at 25 °C and 50% RH. The specimens were initially isothermally cooled for 10 min at -100 °C with liquid nitrogen, before a temperature sweep from -100 to 130 °C with a heating rate of 5 °C min^{-1} was conducted. Rectangular shaped samples (10 mm × 5.3 mm × 0.11 mm) were cut with a stencil and the thickness was measured with a micrometer (IP 65, Mitutoyo). DMA measurements on olive cuticles with and without waxes were carried out using the same protocol, but in a temperature range of -100 to 150 °C and a 3 °C min^{-1} heating rate. To determine the effect of water on the mechanical properties of the cuticles, we used a temperature range of 10 – 40 °C to avoid water freezing and aliphatic wax melting. Due to their fragility, the samples were mounted dry, measured, and then sprayed *in situ* with water. The specimens were allowed to equilibrate for 10 min, before excess water was wicked off and their mechanical properties were measured under wet conditions. All thermal transition temperatures were determined from the maxima of the damping factor tanδ (i.e., loss to storage modulus ratio, also known as loss tangent) curves. Note that in the case of olive cuticles, the presence of cuticular pegs and especially trichomes, leads to an overestimation of the thickness measured with a mechanical micrometer, a subsequent overestimation of the cross-sectional area as previously highlighted[5], and therefore a systematic underestimation of the storage modulus *E'* values. Given that the trichome-free cuticular regions, which constitute more than 80% of the surface (see Supplementary Note 13), display a thickness of ca. 10 μm (native cuticles: 7 ± 1 μm, wax-free cuticles: 8 ± 1 μm, n = 15 using 5 different TEM images; alternatively, native cuticles: 12 ± 1 μm, n = 5 using the "gravimetric method" and a density of 1 g cm^{-3} [5,6]) and the thickness values determined with a micrometer were ca. 30 μm (native

cuticles: 29 ± 3 µm, wax-free cuticles: 26 ± 4 µm, n = 15 from 5 different membranes), the reported storage modulus *E'* values are underestimated by a factor of ca. 2 – 3.

Supplementary Note 4. Wide-angle X-ray scattering (WAXS)

Wide-angle X-ray scattering (WAXS) patterns were collected for the neat SBS and CNCs at room temperature using a NanoMax-IQ camera (Rigaku Innovative Technologies, Auburn Hills, MI, U.S.A.) at room temperature. The collected 2D SAXS patterns were azimuthally integrated and the resulting 1D data ($I(q)$ vs q) were converted to spectra of $I(\theta)$ vs θ with q corresponding to the scattering vector ($q = 4\pi\lambda^{-1}\sin(\theta/2)$), where θ and $\lambda = 0.1524$ nm are the scattering angle and X-ray wavelength, respectively.

WAXS spectra for a neat SBS membrane and freeze-dried CNCs were used to investigate the presence of ordered structures (Supplementary Figure 2e). For the case of CNCs, the characteristic lattice diffraction peaks of semi-crystalline cellulose I were detected[7]. The relative degree of crystallinity (*C.I.*) of CNCs was estimated by using the intensity-method of Segal[8] with Equation (5):

$$C.I. = \frac{I_{002} - I_{\mathrm{AM}}}{I_{002}} \tag{5}$$

where I_{002} corresponds to the intensity of the 002 lattice diffraction peak located at ~22.3° and I_{AM} corresponds to the intensity of the "amorphous" (less-ordered regions) contribution of the sample located at ~18°. Using this method, the crystallinity index (*C.I.*) of CNCs was determined to be 82%. In the case of SBS, well-defined diffraction peaks were absent and a broad amorphous halo was detected. These observations are in agreement with previously reported spectra for SBS[9].

Supplementary Note 5. Conductometric titration of CNCs for the determination of half-sulfate ester groups

Conductometric titration was performed with a conductivity meter (SevenMulti pH Conductivity Meter, Mettler Toledo) to determine the concentration of half-sulfate ester groups that form during the hydrolysis of cellulose. Freeze dried CNCs were redispersed in ultra pure water (arium® pro, Sartorius) at a concentration of 0.5 wt%. The suspension was passed through a column packed with a strong acid cation resin (Dowex® Marathon™ C hydrogen form, Sigma-Aldrich) to protonate the surface of the

CNCs according to previously reported procedures[10]. An aqueous solution of NaOH (0.01 M) was used to titrate the suspension of CNCs and the conductivity was recorded as a function of added volume of NaOH. The concentrations of sulfate half-ester and weak acid groups were determined by two linear regressions on the volume-corrected conductivity data. The concentration of sulfate half-ester groups was determined to be 254 mmol kg^{-1} from the first inflection point of the titration (Supplementary Figure 2f), which is close to previously reported values[10]. The second inflection point corresponded to weaker acid groups (perhaps carboxyl groups -COOH)[11] and was calculated to be 56 mmol kg^{-1} of CNCs.

Supplementary Note 6. Determination of the colloidal stability of CNCs in THF by UV-Vis spectroscopy

Ultraviolet–visible (UV-Vis) spectroscopy was conducted on a Jasco V-670 spectrophotometer at 50 °C using 10 mm path length quartz cuvettes to observe the sedimentation of CNCs in THF as a function of time (Supplementary Figure 3b). Freeze dried CNCs were redispersed in THF at the highest concentration that was used to prepare the membranes (0.3 wt%) by stirring for 30 min and sonicating for 2 h in a sonication bath (Sonoswiss SW3H ultrasonic bath) at room temperature. Pure THF was used as a baseline. The UV-Vis spectra were acquired in transmission mode in time intervals of 1 minute over the course of 60 minutes. We monitored the development of the transmission at 600 nm, a typical wavelength for turbidity experiments with nanocellulose[12,13] to avoid interference and absorption phenomena. Initially, the transmitted light at 600 nm was low (~10%) due to excessive scattering by the CNCs (Supplementary Figure 3b). However, CNCs are known to partially aggregate and sediment in THF[14-18] and this effect manifested itself by an increase of the transmission over time due to attenuated scattering (Supplementary Figure 3b).

Supplementary Note 7. Imaging of the nanocomposite membranes with polarized optical microscopy (POM)

Polarized Optical Microscopy images of SBS/CNC membranes with a thickness of ~ 55 μm (Supplementary Figure 3d-g) were acquired using an Olympus BX51 equipped with a DP72 digital camera in transmission mode with a 20x objective lens under crossed polarizers. Optical anisotropy was

evident in all the acquired images of the nanocomposites (Supplementary Figure 3e→g) whereas birefringence was absent in the case of the neat SBS membranes (Supplementary Figure 3d). The birefringent domains were more pronounced with increasing CNC-content. In all the tested composite membranes presenting optical anisotropy, a uniform appearance of bright birefringent areas was observed, indicative of homogeneously distributed particles within the plane of the membranes.

Supplementary Note 8. Water uptake measurements

Water uptake (i.e., water sorption or swelling) experiments were conducted to determine the equilibrium water uptake of the membranes at 25 °C. The SBS/CNC nanocomposite membranes (diameter of 7.2 mm and ~120 μm thickness) were dried in a vacuum oven at 70 °C for 24 h and afterwards cooled in a desiccator. The initial dry (M_0) and equilibrium wet weight (M_∞) of the membranes after immersing them in DI water (100% RH) or placing them in a closed oven with steady RH conditions (85 or 75%) were determined gravimetrically after > 24 h. The water-immersed membranes were gently blotted with filter paper before measuring their weight. The equilibrium water uptake was calculated as follows:

$$\text{Water uptake (\%)} = \frac{M_\infty - M_0}{M_0} \times 100 \tag{6}$$

The measurements were carried-out in triplicate (n = 3, different samples) for each composition. Reliable gravimetric sorption and desorption kinetic studies of water could not be conducted with this method due to the geometry of the samples and the relatively rapid and low water sorption of the specimens. Therefore, only equilibrium water uptake values were calculated and reported. For the cuticular membranes, their water uptake was determined instead at 97% RH and 25 °C using an oven and a saturated aqueous solution[4] of K_2SO_4 due to their fragility. The water uptake was 6.1 ± 2.4 % for the wax-containing and 6.9 ± 1.3 % for the wax-free olive cuticles at 97% RH.

Supplementary Note 9. Diffusion of water using *in situ* ATR-IR spectroscopy

The diffusion of water through the nanocomposite membranes was investigated using a PerkinElmer Spectrum 65 spectrometer in Attenuated Total Reflectance (ATR) mode (Universal ATR model, ~1.7 μm depth of penetration) following a procedure proposed previously by Fieldson and Barbari[19]. With

this method, diffusion coefficients, D, can be determined by applying a sample on the surface of an ATR-IR crystal followed by deposition of the penetrant on the opposite side of the membrane and monitoring the evolution of the absorbance corresponding to IR-active species such as water (e.g., –OH group regime) over time. Thus, the top side of the nanocomposite membranes (pre-dried for 16 h in a vacuum oven at 70 °C and subsequently stored in a desiccator) faced the ATR crystal and was fixed with a mechanical clamp. For the opposite configuration (i.e., the bottom side of the membranes facing the ATR crystal), reliable tracking of the water –OH signal was not possible, as the –OH signal of the CNCs interfered with this signal. DI water was applied on CNC rich bottom side of the membranes (~ 33 μm thickness) and IR spectra were immediately recorded between 4000 and 2500 cm^{-1} in absorbance mode at a resolution of 16 cm^{-1}, 40 number of scans (64 s per spectrum) and at a temperature of 22 °C. The acquired spectra were baseline corrected using a spectroscopy software (Spectrum10, PerkinElmer) followed by integration of each spectrum at the 3680-3050 cm^{-1} region with Origin (OriginLab). The integrated region is proportional to the amount of water diffused through the membranes and the diffusion coefficient of water was determined by fitting the evolution of the integrated signal (A) with time (t) using Equation 7. The value of A_∞ was determined by regression of the raw data instead of a direct point measurement as previously reported[19]. For each composition, at least two membranes ($n \geq 2$) were used to determine the apparent diffusion coefficient D.

$$\ln\left(1 - \frac{A}{A_\infty}\right) = \ln\left(\frac{4}{\pi}\right) - \frac{D\pi^2}{4\ell^2}t \tag{7}$$

Note that the diffusion coefficient of the composite membranes was inversely proportional to the CNC content (Supplementary Figure 8c), which is consistent with the highly crystallinity nature of the CNCs (Supplementary Figure 2e), whose integration into the fully amorphous SBS increases the diffusion pathways by increasing the tortuosity of the membrane. In spite of this, the water permeability followed an inverse behavior and increased monotonically with the CNC content upon exposure of the CNC-rich bottom side of the composites towards the water-containing donor compartment of the gravimetric cup method tests (main text Figure 2c), whereas the inverse test (i.e., top side – poor in CNCs towards the donor) showed negligible differences compared to the neat membranes (main text Figure 2d).

Supplementary Note 10. Static water contact angle measurements with sessile drop method

Static water contact angle measurements were conducted by using a contact angle setup (Contact Angle System OCA 15 Pro, Dataphysics, Germany) with the sessile drop method at room conditions. A small droplet (5 μL) of DI water was applied with a needle on the surface of the membranes and an integrated camera was utilized to observe the water droplet after 30 s. Baseline establishment and contact angle determination were performed with the software SCA 20 (Dataphysics). At least five droplets were used to determine the mean contact angle and the standard deviation of the samples. The wetting properties of both sides of the membranes were tested with samples pre-equilibrated overnight in DI water and blotted with lint-free lab tissues.

Water contact angle measurements were carried out on both sides of SBS/CNCs 15 wt% and neat SBS to investigate the effect of CNCs on the wetting properties of the membranes (Supplementary Figure 8d). A decrease of approximately 9° was observed for the rich in CNCs bottom side of the SBS/CNCs 15 wt% membranes (81 ± 3) induced by the incorporation of the hydrophilic CNCs in the hydrophobic SBS matrix. Conversely, the top side of the same membranes presented contact angle values identical to the neat polymer (90 ± 2), which was in agreement with the acquired ATR-IR data (Supplementary Figure 4). The contact angle determined for the neat SBS lies within the range of previously reported values[20,21].

Supplementary Note 11. Small-angle X-ray scattering (SAXS)

A NanoMax-IQ camera (Rigaku Innovative Technologies, Auburn Hills, MI, U.S.A.) was used to collect X-ray scattering data and investigate the effect of CNCs on the morphology of the prepared SBS/CNC membranes (ℓ ~ 33 μm). The collected 2D SAXS patterns were radially averaged, thus obtaining 1D SAXS curves of $I(q)$ with q corresponding to the scattering vector ($q = 4\pi\lambda^{-1}\sin(\theta/2)$), where θ and $\lambda = 0.1524$ nm are the scattering angle and X-ray wavelength, respectively) (Supplementary Figure 9d). The SAXS spectra were acquired at room temperature using “as casted” membranes without employing any annealing process to study the impact of CNCs on the block copolymer self-assembly.

All of the SAXS curves presented a well-defined primary peak located at a scattering vector $q^* = 0.19$ nm^{-1} (Supplementary Figure 9d). Therefore, the domain spacing, d, was determined to be approximately 33 nm ($d = 2\pi/q^*$). Weak Bragg peaks were identified in the vicinity of a scattering angle $\sqrt{3}\, q^*$ and $\sqrt{7}\, q^*$ corresponding to a hexagonally packed cylinders morphology as previously reported for similar compositions of SBS[22,23]. The addition of CNCs in the matrix seemed to have a negligible effect on the phase segregation of the block copolymer.

Supplementary Note 12. Procedure for determining the effective membrane area of the inner cuticular side

The enzymatic isolation of the cuticular membranes from the cell wall featured bound cell wall matter (Supplementary Figure 1g and h, Supplementary Figure 10), known as cuticular pegs[24,25], on the inner cuticular side of the membranes that cannot be removed enzymatically, mechanically or chemically without affecting the fragile cuticular architecture. These protrusions increase the available sorption area as previously speculated for cuticular membranes isolated from leaves or fruits of other species (*Citrus, Ficus, Lycopersicon* and *Capsicum*)[26]. As a result, the effective membrane area of the inner cuticular side (A_{ICS}) of olive (*Olea europaea*) leaf cuticles is larger than that of the respective outer cuticular side (A_{OCS}). We estimated the A_{ICS} to be 1.54 ± 0.14 times larger than the A_{OCS} (Supplementary Figure 10a). Using SEM stereopair images (n = 5 images) of 136 residual cells and the image processing software ImageJ 1.51k, we determined the number of residual cells N_c in each image (non-overlapping with the edges), the mean cell perimeter (P_c = 56 ± 2 μm) and area (A_c = 203 ± 12 μm^2), and the height (h_c = 3.7 ± 0.9 μm) of the residual cell walls. This calculation resulted in a surface area factor of the inner cuticular side of 1.54 ± 0.14 yielding a mean effective surface of 4.3×10^{-5} m^2 (corrected A_{ICS}). This value of A_{ICS} was used to area-normalize the acquired permeation data when the inner cuticular side was facing the donor compartment, while A_{OCS} (2.8×10^{-5} m^2) was used for the opposite direction experiments. In contrast to olive cuticles, the inner side cuticular pegs of ivy (*Hedera helix)* leaf cuticles are less pronounced (Supplementary Figure 10d-e) and, therefore, no surface area correction was applied in their case. Similar observations for the relatively flat inner cuticular side of ivy cuticles have been previously reported[25].

Supplementary Note 13. Verification of transversal heterogeneity of cuticles

The asymmetric structure and composition of olive leaf cuticles are well known from previous studies[27-31] and, therefore, an exhaustive analysis of these properties was omitted. To confirm the cuticular transversal heterogeneity, we used ATR-IR spectroscopy to highlight the compositional contrast between the inner and outer cuticular side at a surface level (~1.7 μm penetration depth) (Supplementary Figure 1a-b) and electron microscopy to image their cross-section (Supplementary Figure **1**c-f). As demonstrated by the ATR-IR spectra acquired on the two sides of olive cuticles (Supplementary Figure **1**a-b), the inner cuticular side is notably richer in polar functional groups associated with polysaccharides (e.g., –OH and C-O/C-C at ~3360 cm^{-1} and 1030 cm^{-1}, respectively) (see also Supplementary Table 2) in comparison to the outer cuticular side. Moreover, relatively non-polar functional groups are also stronger towards the outer cuticular side as for example the CH_2 stretching vibrations at 2918 and 2850 cm^{-1} (Supplementary Figure 1a-b, Supplementary Table 2). Upon wax-removal (Supplementary Figure 1b), except for the notable elimination of vibration bands associated with phenolic compounds and triterpenoids (e.g., C=O and C=C of triterpenoids and phenolics at 1686 and 1546 cm^{-1}, respectively), the outer cuticular side of the wax-free olive cuticles is richer in carbonyl (C=O at 1731 cm^{-1}) and ester (O=C-O-C at 1164 cm^{-1}) groups (see also Supplementary Table 2), which are indicative of a higher cutin content compared to the inner cuticular side[32]. Thus, the acquired ATR-IR spectra on the two sides of the membranes confirmed the transversal heterogeneity of olive cuticles. Similarly to the olives, the ivy cuticles also demonstrate a compositionally asymmetric transversal architecture (Supplementary Figure 11), in agreement with previous reports[24].

To further investigate the asymmetric transversal structure of olive cuticles, we also acquired images of their cross-sections using TEM and FIB-SEM (Supplementary Figure 1c-f). Three main layers are detected across the olive cuticle cross-section: (i) a gradually fading reticulate zone towards the inner cuticular side that according to ATR-IR (Supplementary Figure 1a-b) and literature data[24,28,29,33,34] consists mainly of polysaccharides, (ii) an intermediate inner cuticular layer that is presumably mainly composed of cutin and fading polysaccharide fibrils[28,29], and (iii) an outer cuticular layer (also known as cuticle proper), which has been reported to be an amorphous, rich in cutin and intracuticular waxes

region[24,33,34] (Supplementary Figure 1c-f). Towards the outer cuticular side, a lamella-free (Supplementary Figure 1c,e), but also in rare cases (<5% of the length of the TEM sections) a lamellate morphology (Supplementary Figure 1d,f), were observed in TEM and FIB-SEM images. Thus, the dominant morphology of olive leaf cuticles is the lamella-free architecture as previously suggested[28,29]. Following Holloway's structural classification of cuticles[24], cuticular membranes isolated from the adaxial side of olive leaves belong in the family of Type 1-3 cuticles (i.e., faintly reticulate, mainly amorphous, but also locally lamellated) and ivy membranes are Type 2 cuticles.

Trichomes were also observed on the outer cuticular side of olive cuticles, covering 18 ± 3 % of the total outer cuticular area (analysis of n = 9 SEM images in total from n = 3 different samples using the image processing software ImageJ 1.51k) (Supplementary Figure 1g-h, Supplementary Figure 13). These trichomes, also known as peltate hair or scales, cover both the abaxial (lower) and adaxial (upper) side of olive leaves, but are much more abundant at the stomata-containing abaxial side[35,36]. The trichomes of olive cuticles are postulated to protect the underlying tissues by absorbing UV-irradiation[37,38], among other functions[35] and, in the case of adaxial side olive leaf cuticles (as in our work), they are not actively participating in the uptake of water[39], which —in contrast— is postulated to be the case for other drought-resistant plants that rely on trichomes for water uptake[40]. Indeed, the detected water permeances through isolated olive cuticles in this work are indicative of a typical dense membrane ($2 \times 10^{-10} – 1 \times 10^{-8}$ m s^{-1}, for comparison neat SBS membranes of a 33 μm thickness have a permeance of ca. 1×10^{-9} m s^{-1}, Supplementary Tables 3 – 5) irrespective of the utilized conditions (humidity, exposure side) in agreement with previous studies suggesting a similar dense membrane transport behavior[30,41]. Using TEM and FIB-SEM, we observed that a continuous dense cuticle is present underneath the trichomes (Supplementary Figure 13). This cuticular layer functions as a mass transfer-limiting dense membrane irrespective of the transport directionality, since small molecules such as water have to sorb and diffuse through this non-porous layer.

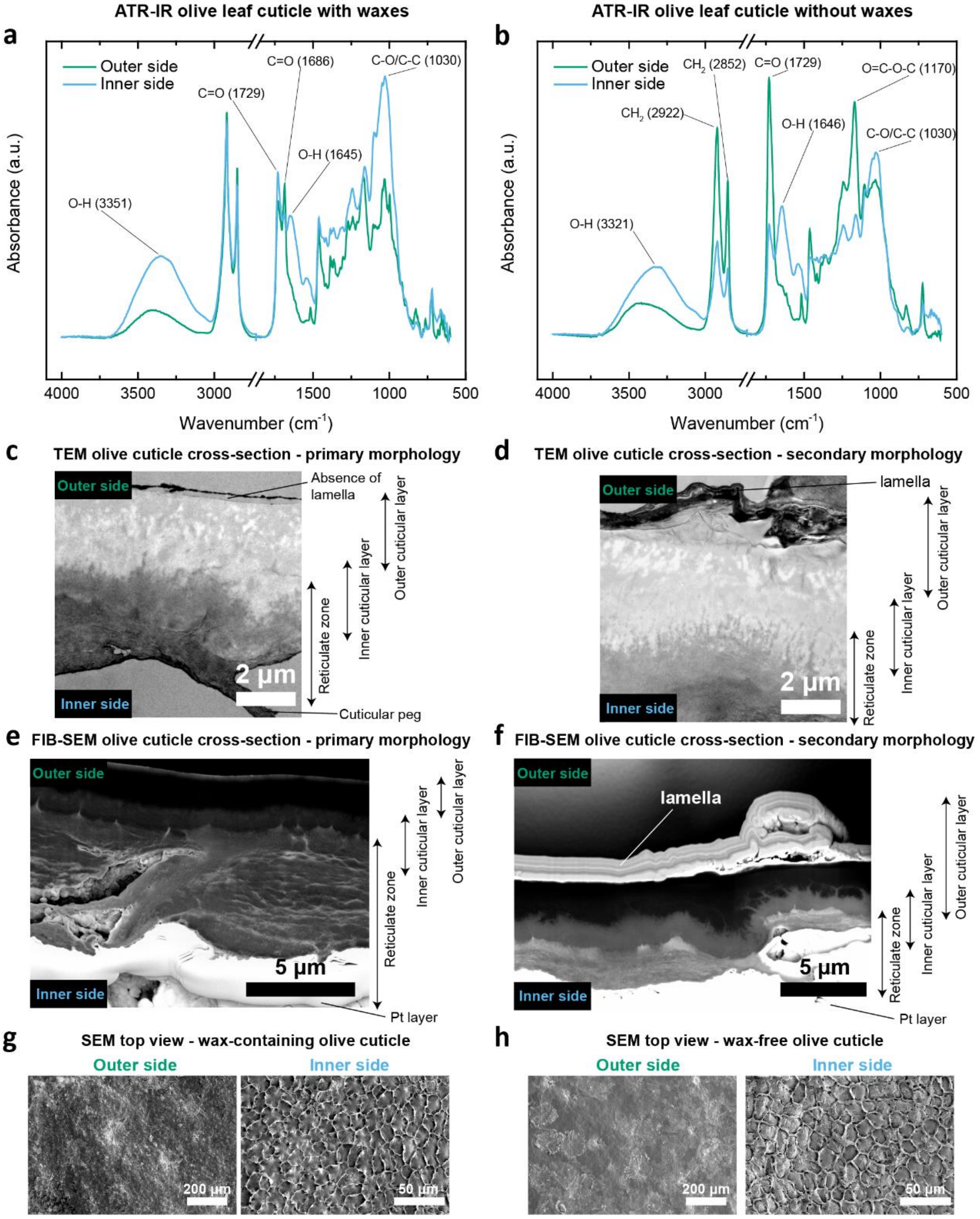

Supplementary Figure 1. Asymmetric architecture of olive cuticles. (a-b) ATR-FTIR spectra of enzymatically isolated adaxial side olive leaf cuticles (a) with waxes and (b) without waxes of the physiologically inner and outer cuticular side. The ATR-IR spectra demonstrate a relatively larger polysaccharide content at the inner side as evidenced by the stronger bands associated with polysaccharides, whereas groups associated with waxes and cutin are more prominent towards the outer side (see bands analytically at Supplementary Table 2). (c-d) TEM and (e-f) FIB-SEM images showing the cross-section of wax-containing enzymatically isolated olive cuticles. Note that

the FIB-SEM images were acquired at an angle of 38° and the bright white layer at the inner cuticular side corresponds to the sacrificial protective layer of Pt deposited prior to milling of the samples using gallium ions. Three distinct layers are distinguishable: (i) a polysaccharide-rich reticulate zone towards the inner cuticular side, (ii) an intermediate inner cuticular layer rich in polysaccharides and cutin, and (iii) an outer cuticular layer (also known as cuticle proper) rich in cutin and waxes at the outer cuticular side. The outer cuticular layer of olives is primarily non-lamellate, although lamellate structures are also present locally (see also discussion at Supplementary Note 13). (g-h) Top-view SEM images of olive cuticles (g) with waxes and (h) without waxes at the outer and inner cuticular sides.

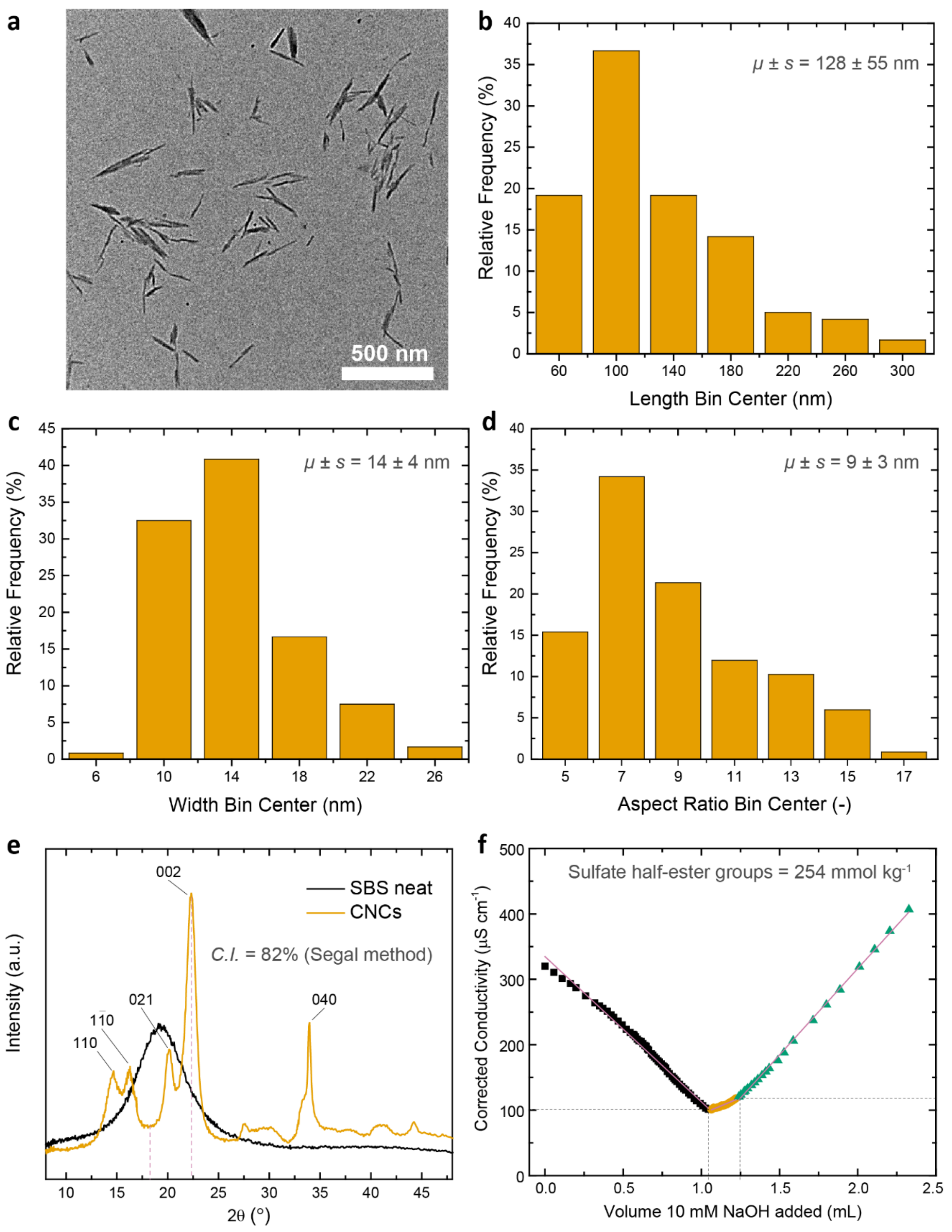

Supplementary Figure 2. (a) TEM image of cotton-derived CNCs deposited from a 0.03 wt% aqueous dispersion. (b-d) Relative frequency histograms of the length (b), width (c), and aspect ratio (d) of the CNCs used for size analysis. Five different images with a total of 120 CNCs were analyzed and the reported values are the mean ± s.d. (e) WAXS diffractograms of the neat SBS and the CNCs measured at 25 °C. (f) Conductometric titration curve of an aqueous suspension of CNCs (~0.4 wt%) using 10 mM NaOH to determine the amount of sulfate half-ester groups introduced during the isolation process of CNC with sulfuric acid. Please refer to Supplementary Notes 1 and 5 for the corresponding calculations and reported values.

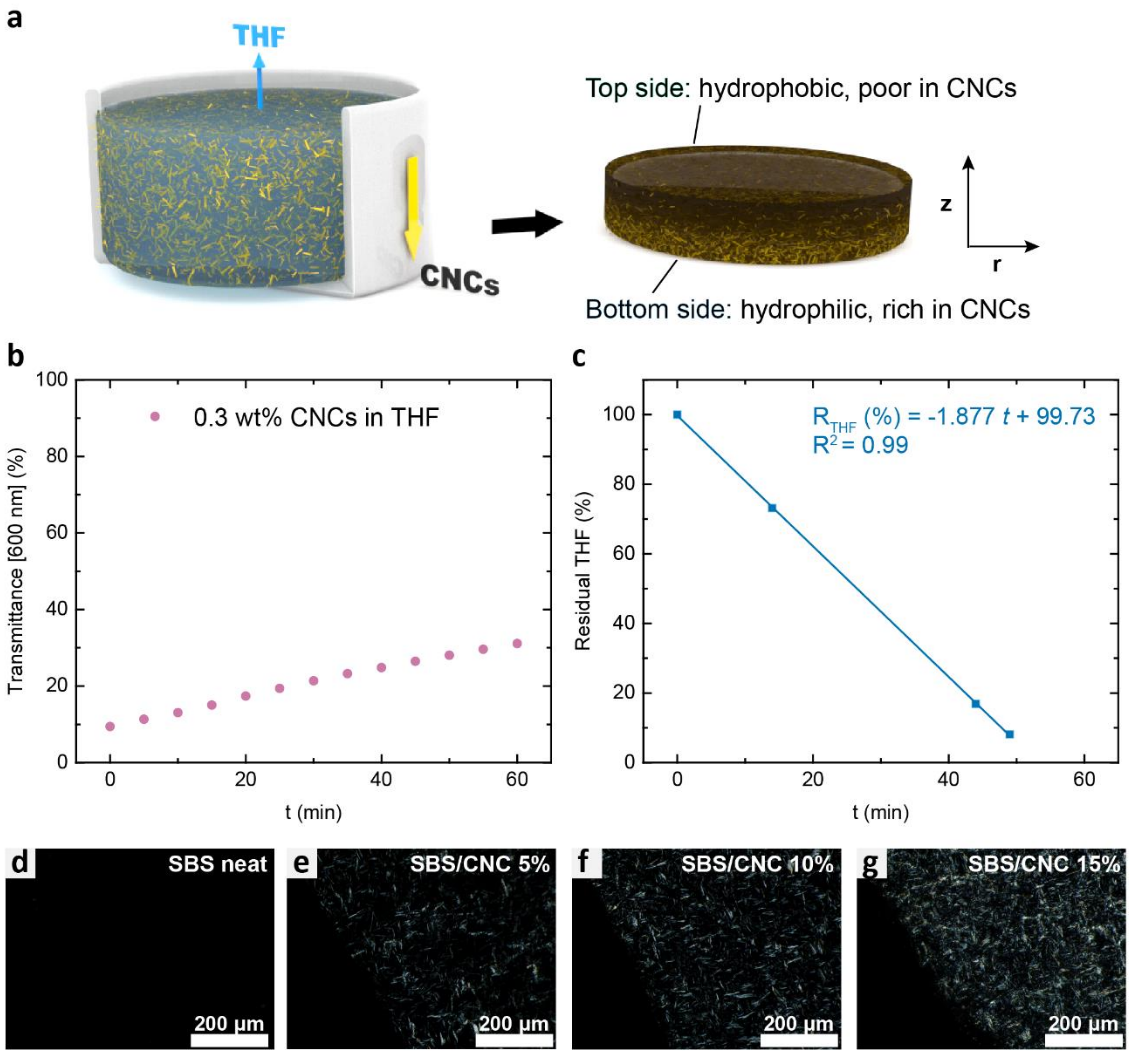

Supplementary Figure 3. Fabrication of SBS/CNC membranes with transversal compositional gradients. (a) Schematic (not to scale) of the solvent casting-evaporation process. (b-c) Graphs reflecting two essential effects during processing. (b) The gravitational sedimentation of CNCs in tetrahydrofuran (THF) (0.3 wt%) was monitored via the transmittance (600 nm) at 50 °C as a function of time. (c) The evaporation of THF from a PTFE dish, which leads to a viscosity increase and solidification, was monitored at 50 °C as a function of time (area normalized initial mass of THF ~ 0.26 g cm^{-2}). (d-g) Polarized optical microscopy images of (d) neat SBS, (e-g) SBS/CNC nanocomposites with 5, 10, or 15 wt% CNCs at 20x magnification displaying homogeneously distributed birefringent domains across the main planes (r-axis) of the CNC-containing nanocomposites.

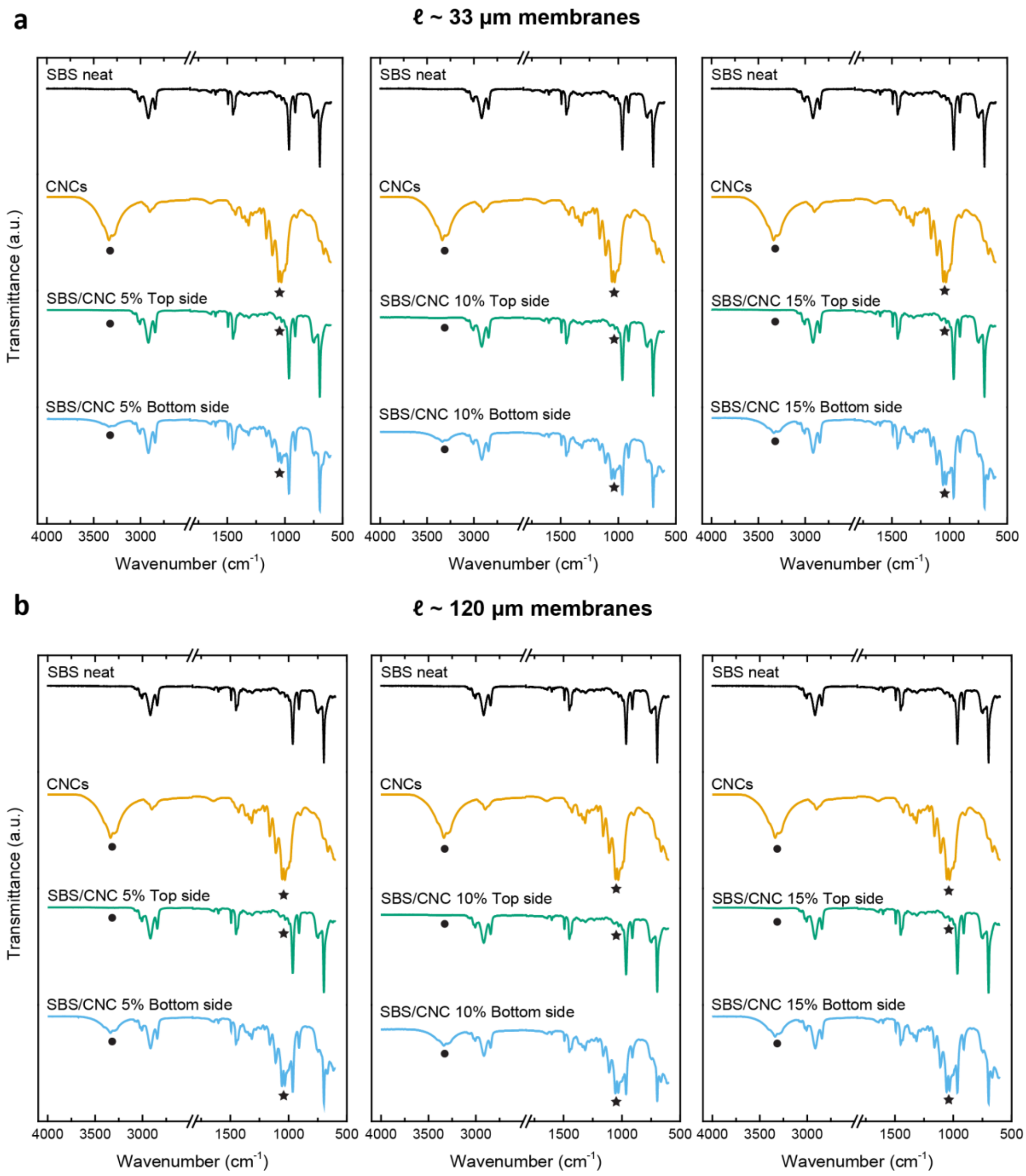

Supplementary Figure 4. Investigation of the transversal compositional gradients of SBS/CNC membranes by ATR-IR spectroscopy. (a-b) ATR-IR spectra of SBS, CNCs, and the SBS/CNC nanocomposite membranes with a thickness (a) ~33 µm and (b) ~120 µm. The spectra acquired on the bottom side of the membranes clearly show signals associated with CNCs at ★ 3338-3288 cm^{-1} and • 1054-1031 cm^{-1}, corresponding to –OH and C-O/C-C bands, but these signals are absent in the spectra acquired on the top side of the membranes.

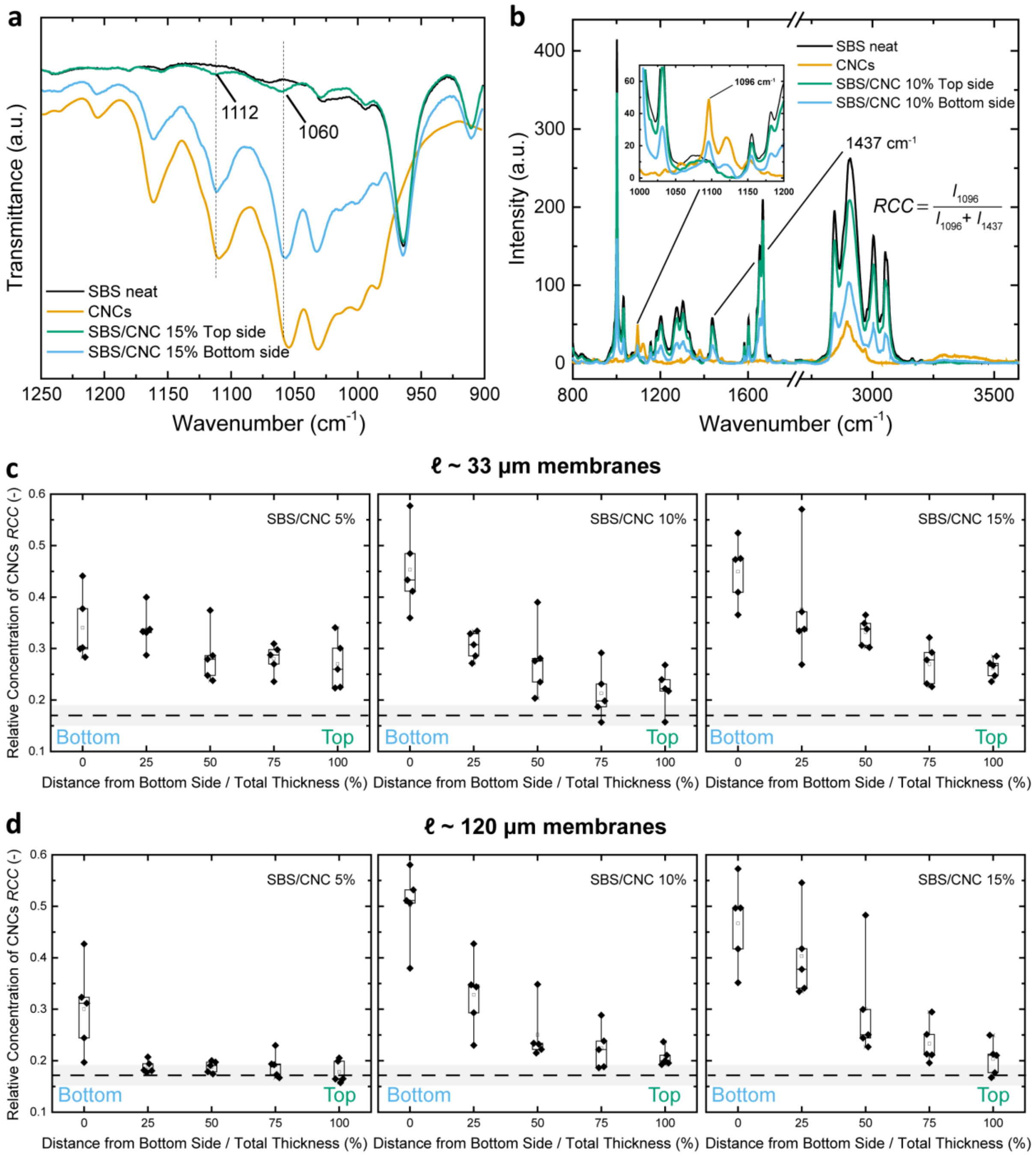

Supplementary Figure 5. Investigation of the transversal compositional gradients of SBS/CNC membranes by ATR-IR spectroscopy and Raman microscopy. (a) ATR-IR spectra of a SBS/CNC nanocomposite membrane containing 15 wt% CNCs show weak vibration bands of CNCs on the top side of the membrane (1112 cm^{-1}, glucose ring stretch; 1060 cm^{-1}, C-C/C-O stretch of CNCs), whereas these signals are pronounced on the bottom side. (b) Raman spectra of neat materials (SBS, CNCs) and of a 120 µm thick SBS/CNC nanocomposite membrane with a CNC content of 10 wt% display bands associated with the CNCs (stretching vibration of C-O: 1096 cm^{-1}) and SBS (CH_2 deformation: 1437 cm^{-1}). (c-d) Raman mapping box charts of SBS/CNC nanocomposite membranes with a thickness (c) ~33 µm and (d) ~120 µm show the evolution of the relative CNC concentration (*RCC*) in the

transversal membrane direction. The *RCC* was calculated from the intensities (*I*) of the CNCs' C-O (1096 cm^{-1}) and SBS' CH_2 (1437 cm^{-1}) vibration bands using n = 5 measurements for each position at different spots. The dashed lines and the grey areas correspond to the mean ± s.d. of the *RCC* of SBS (n = 3 in each position). In all box charts, whiskers extend to min and max values, box edges show 25 – 75 percentiles, center line represents median, hollow square represents mean.

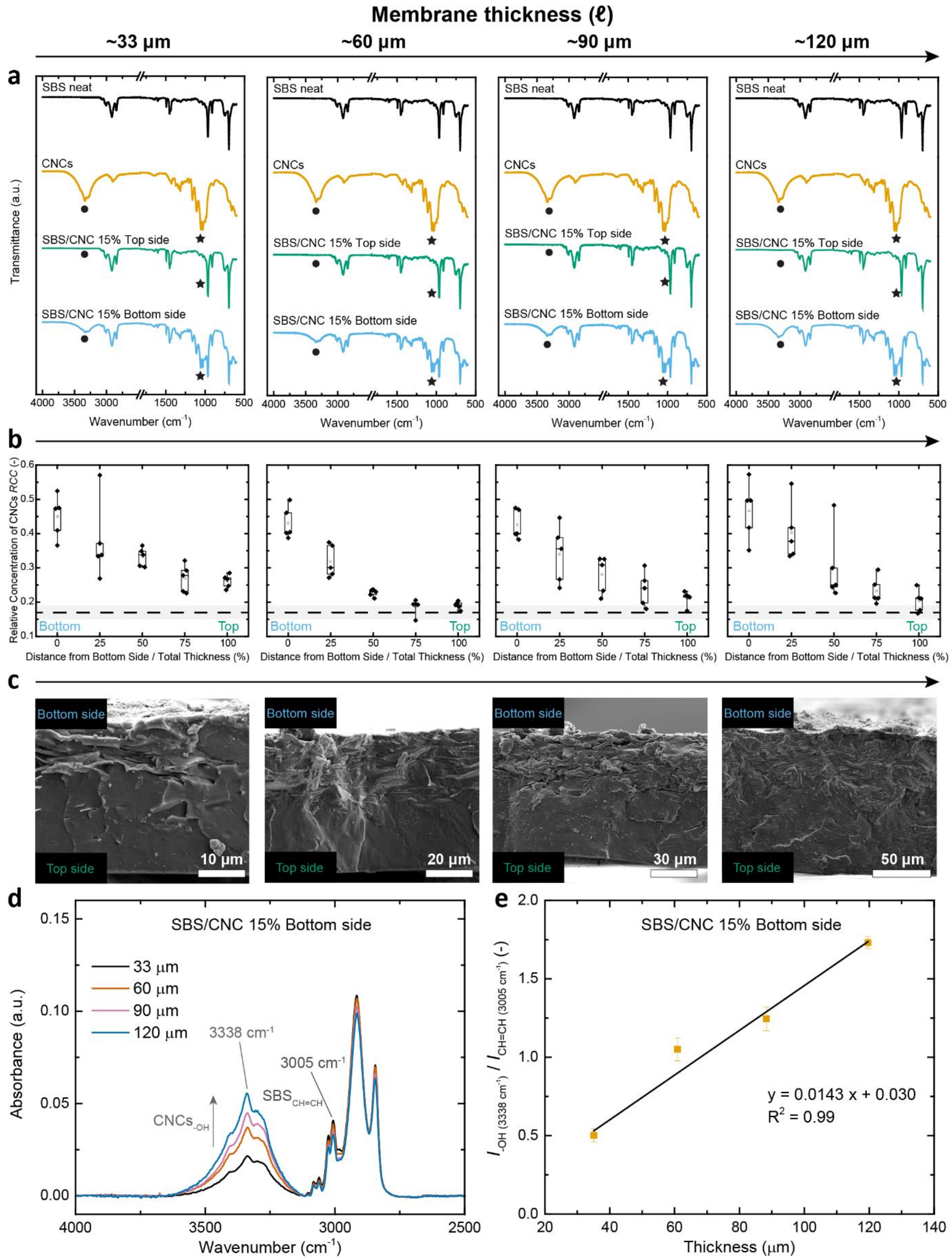

Supplementary Figure 6. Investigation of the transversal compositional gradients of SBS/CNC membranes with 15 wt% CNCs and varying membrane thickness (a) ATR-IR spectra acquired on the bottom sides of the membranes clearly show signals associated with CNCs at ★ 3338-3288 cm^{-1} and •1054-1031 cm^{-1}, corresponding to –OH and C-O/C-C bands, but these signals are absent in the spectra of the top side of the membranes. (b) Raman mapping

box charts showing the evolution of the relative CNC concentration (*RCC*) in the transversal membrane direction. The *RCC* was calculated from the intensities (*I*) of the CNCs' C-O (1096 cm^{-1}) and SBS' CH_2 (1437 cm^{-1}) vibration bands using n = 5 measurements for each position at different spots. Whiskers extend to min and max values, box edges show 25 – 75 percentiles, center line represents median, hollow square represents mean. The dashed lines and the grey areas correspond to the mean ± s.d. of the *RCC* of SBS (n = 3 in each position). (c) SEM images of the cross-sections (note that the membranes are upside down in the images). (d) ATR-IR spectra of the bottom side demonstrating the increasing CNCs' –OH (3630 – 3125 cm^{-1}, peak at 3338 cm^{-1}) and decreasing SBS' CH=CH (3005 cm^{-1}) bands with thickness. (e) Plot of the ratio of the absorbance intensities *I* of the 3338 and 3005 cm^{-1} IR bands (based on (d)) vs thickness scaling linearly (mean ± s.d. of n = 3 spots) showing an increasing relative content of CNCs with larger membrane thicknesses consistent with a prolonged sedimentation process of CNCs when casting thicker membranes under the same casting conditions.

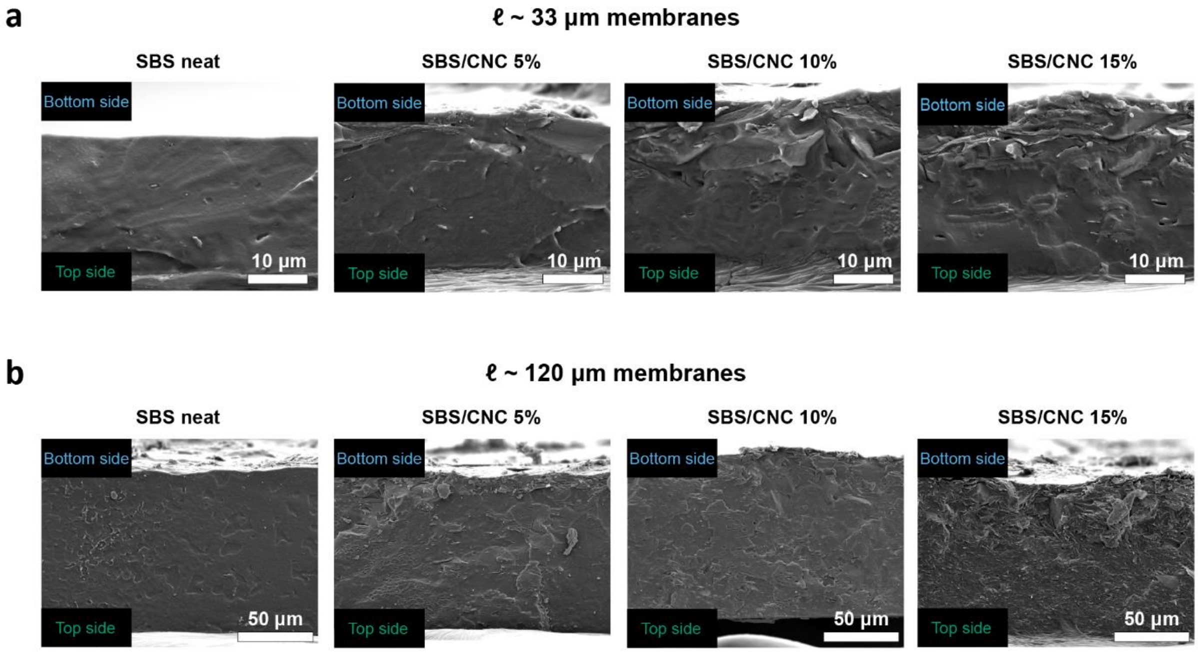

Supplementary Figure 7. SEM imaging of SBS/CNC membranes. (a-b) SEM images of the cross-sections of cryo-fractured SBS/CNC nanocomposite membranes with a thickness (a) ~33 µm and (b) ~120 µm show distinct morphological differences between the bottom and top sides (note that the membranes are upside down in the images).

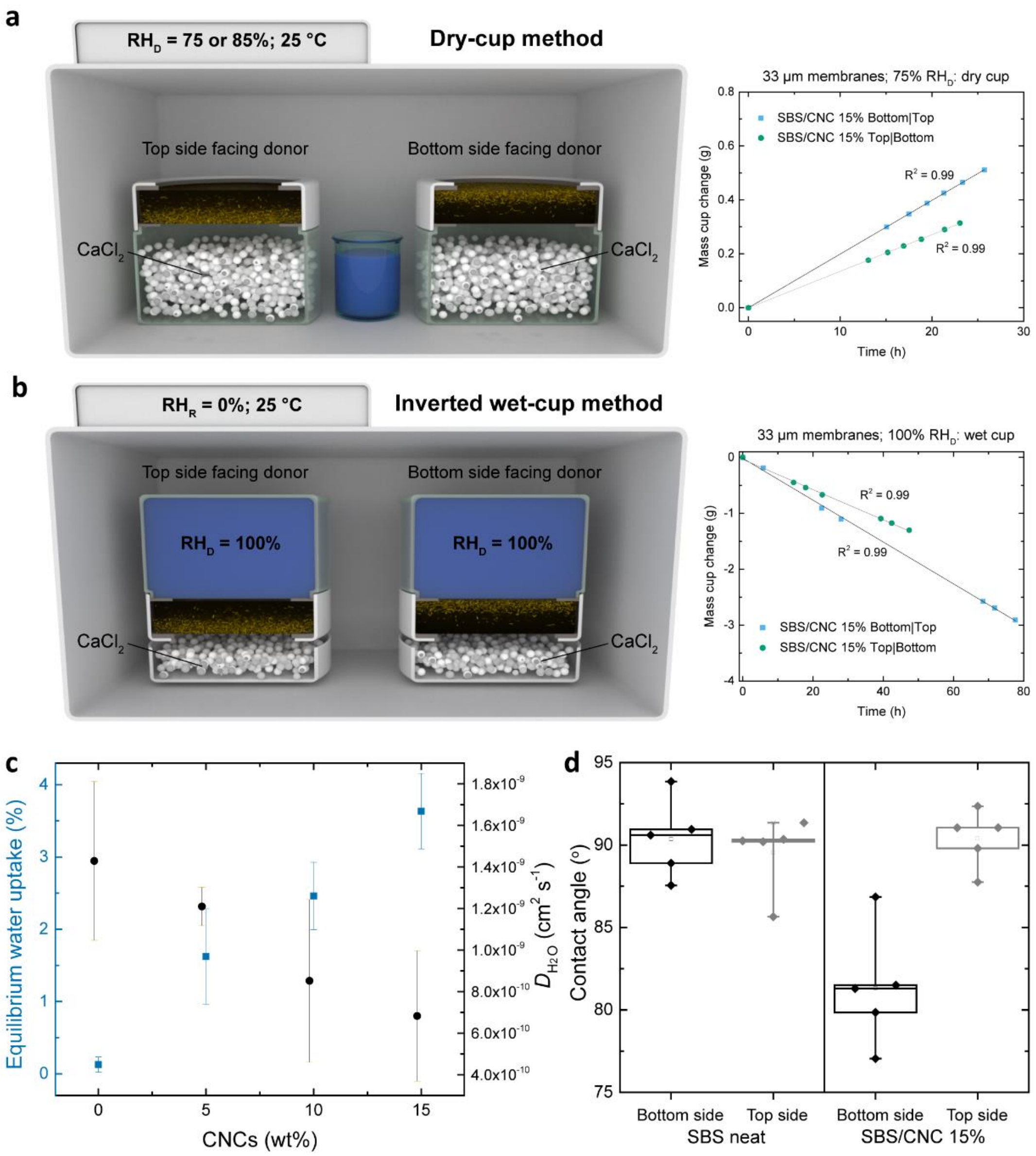

Supplementary Figure 8. Investigation of the water transport, uptake, and contact angle of SBS/CNC membranes. (a-b) Schematic (not to scale) representation of the gravimetric (a) dry cup and (b) inverted wet cup method (ASTM E96) that were used to determine the water permeability from both sides of the SBS/CNC nanocomposite membranes at 75, 85 and 100% RH_D at 25 °C. Representative "mass cup change vs time" curves with the slope ($R^2 > 0.99$) corresponding to the water vapor transmitted (i.e., diffused) through the membranes (ca. 33 µm thickness). (c) Graph demonstrating the evolution of the equilibrium water uptake at 25 °C with samples imbibed in water (squares) and water diffusion coefficient D_{H2O} at 22 °C (circles) determined by *in situ* ATR-IR spectroscopy as a function of CNC content (wt%) in the membranes. The reported data are the mean ± s.d. of n = 3 measurements with different membranes. (d) Box chart showing the water contact angle on both sides of the neat SBS and 33 µm thick SBS/CNC membranes containing 15 wt% CNCs. Whiskers extend to min and max values, box edges show 25 – 75 percentiles, center line represents median, hollow square represents mean.

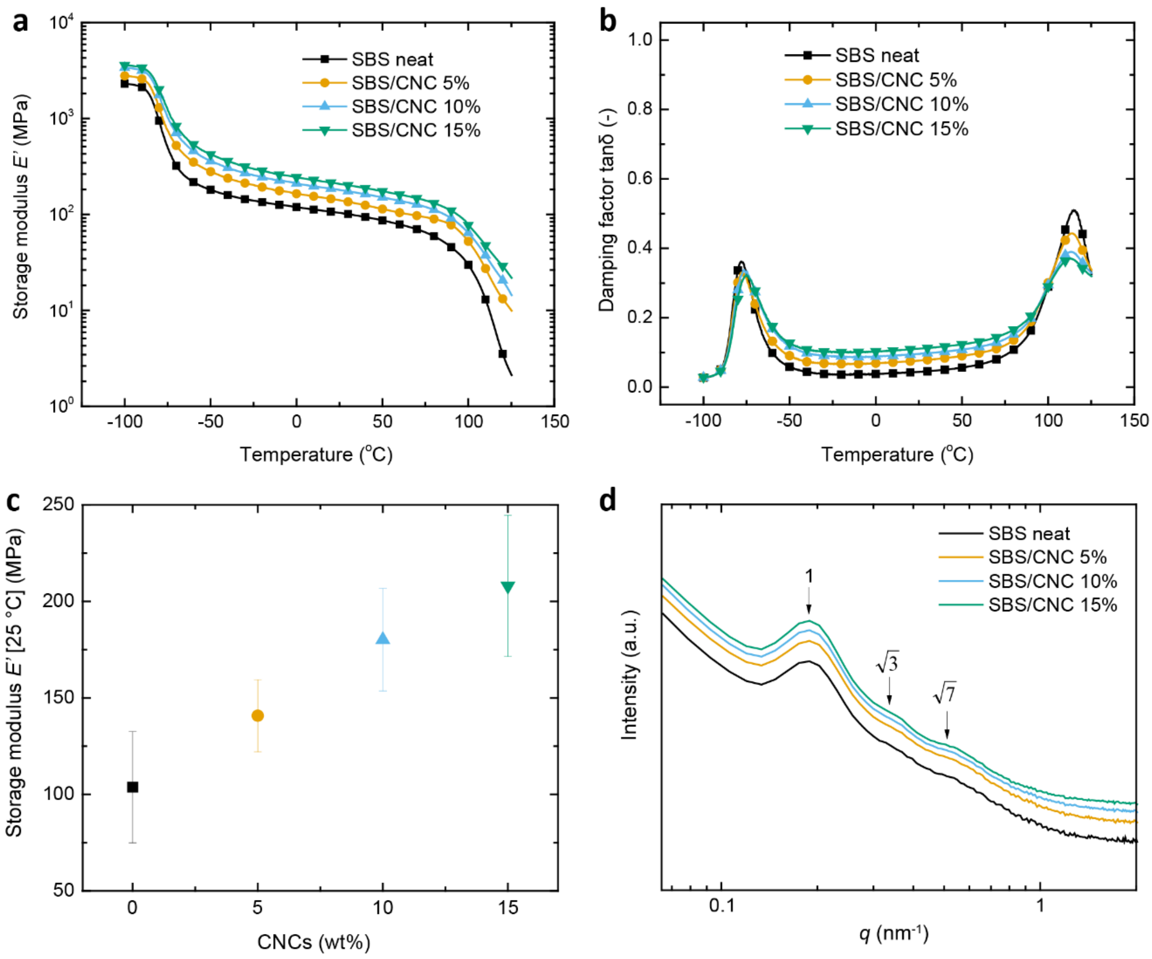

Supplementary Figure 9. Mechanical properties and block-copolymer morphology of SBS/CNC membranes. (a,b) Representative dynamic mechanical analysis (DMA) traces showing (a) the storage modulus *E*' and (b) damping factor tanδ of neat SBS and SBS/CNC nanocomposite membranes containing 5, 10, or 15 wt% CNCs. The data show the presence of two glass transitions (T_g) associated with poly(butadiene) (T_{g1} ~ -78 °C) and poly(styrene) blocks (T_{g2} ~ 113 °C) and a rubbery plateau in between. (c) Plot showing the storage modulus *E*' at 25 °C as a function of CNC content (mean of n = 5 measurements with specimens isolated from one sample for each composition, ± s.d.). (d) Azimuthally integrated logarithmic SAXS spectra of SBS/CNC nanocomposite membranes measured at 25 °C.

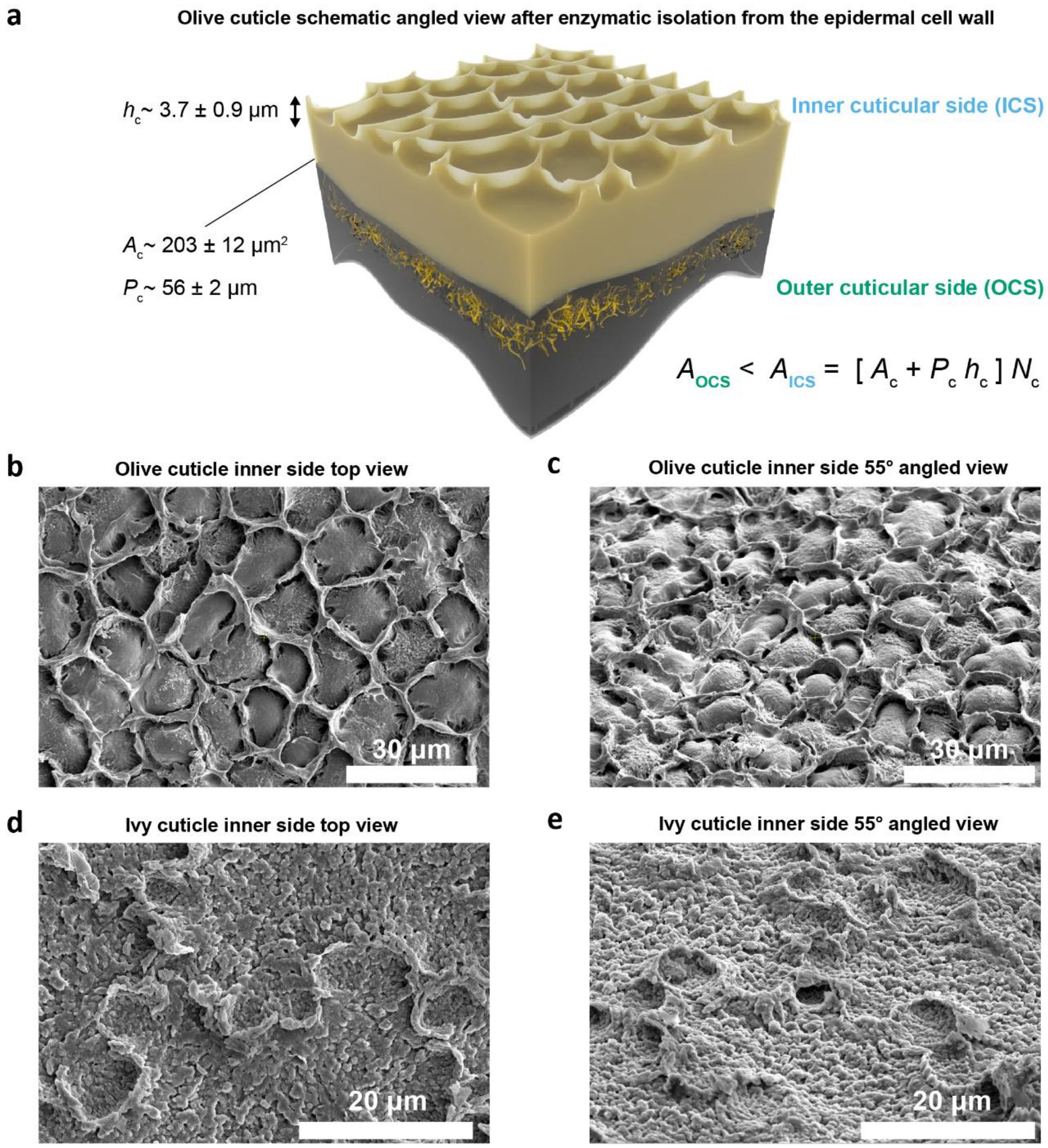

Supplementary Figure 10. Morphology of the olive cuticle inner side and its effect on the effective membrane area. (a) Schematic (not to scale) representation of an olive cuticle's transversal cross-section after enzymatic isolation from the epidermal cell wall. Upon isolation of the cuticle, bound cell wall matter (cuticular pegs) remains on the inner cuticular side (ICS). Thus, the available surface area at the inner cuticular side (A_{ICS}) is larger than that of the flat outer cuticular side (A_{OCS}) To calculate the effective surface area of the inner cuticular side (A_{ICS}), we determined the number of residual cells per image (N_c), the mean cell perimeter (P_c), height (h_c) and area (A_c) using ImageJ 1.51k. (b-c) Representative SEM images of the inner cuticular side (ICS) of olive cuticles: (b) top view and (c) at an angle of 55° to determine (b) the mean perimeter (P_c), area (A_c) and (c) height (h_c) of the bound cell wall matter. (d-e) Representative SEM images of the inner cuticular side (ICS) of ivy cuticles: (d) top view and (e) at an angle of 55° with less pronounced cuticular pegs.

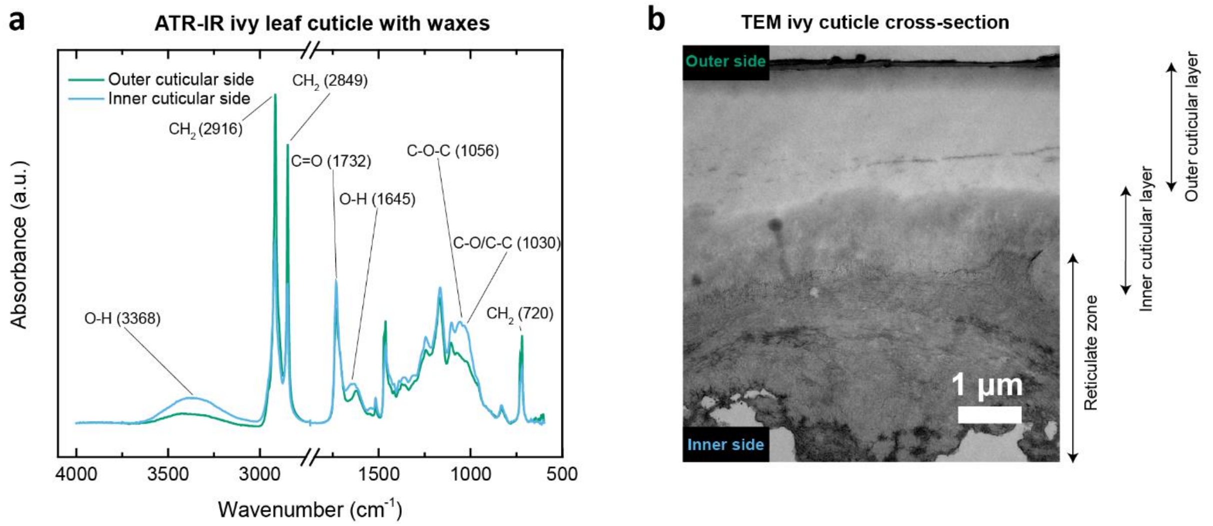

Supplementary Figure 11. Asymmetric architecture of ivy cuticles. (a) ATR-FTIR spectra of enzymatically isolated adaxial side native ivy leaf cuticles (i.e., with waxes) of the physiologically inner and outer cuticular side. The ATR-IR spectra demonstrate a relatively larger polysaccharide content at the inner cuticular side compared to the outer side as evidenced by the stronger bands associated with polysaccharides towards the inner side, whereas less polar groups associated with waxes and cutin are more prominent towards the outer side (see bands at Supplementary Table 2). (b) TEM image showing the cross-section of a wax-containing enzymatically isolated ivy cuticle. Three distinct layers are distinguishable: (i) a polysaccharide-rich reticulate zone towards the inner cuticular side, (ii) an intermediate inner cuticular layer rich in polysaccharides and cutin, and (iii) an outer cuticular layer (also known as cuticle proper) rich in cutin and waxes.

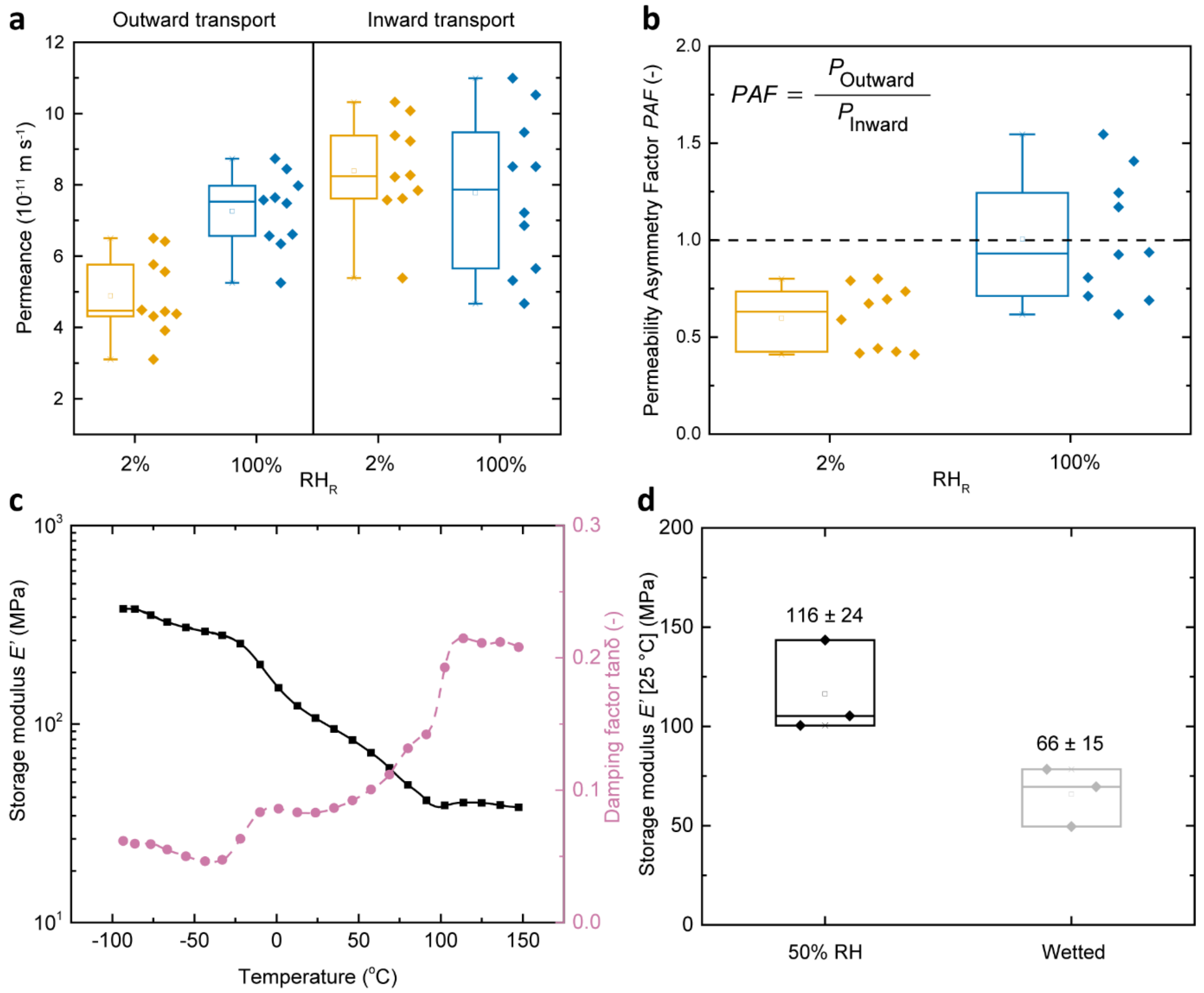

Supplementary Figure 12. Water permeation and thermomechanical properties of ivy (*Hedera helix*) wax-containing leaf cuticles. (a) ^3H_2O permeances at different receiver relative humidity conditions (RH_R = 2 or 100%). The outward and inward transport correspond to the inner and outer cuticular side facing the donor (RH_D = 100%), respectively. (b) Box plot of the Permeability Asymmetry Factor (*PAF*) determined by ^3H_2O permeation experiments (data from (a)) determined as the ratio of outward and inward transport. The experiments were conducted at 25 °C with n = 10 different membranes. (c-d) Dynamic mechanical analysis (DMA) data of ivy cuticles: (c) representative DMA traces of the storage modulus *E'* (solid line) and the damping factor tanδ (dashed line) at a temperature range of -100 to 150 °C, and (d) box chart displaying the storage modulus *E'* of ivy cuticles at 25 °C conditioned at 50% RH and wetted with water showing the plasticizing effect of water. Reported values are the mean ± s.d. of n = 3 measurements (paired samples). In all box charts, whiskers extend to min and max values, box edges show 25 – 75 percentiles, center line represents median, hollow square represents mean.

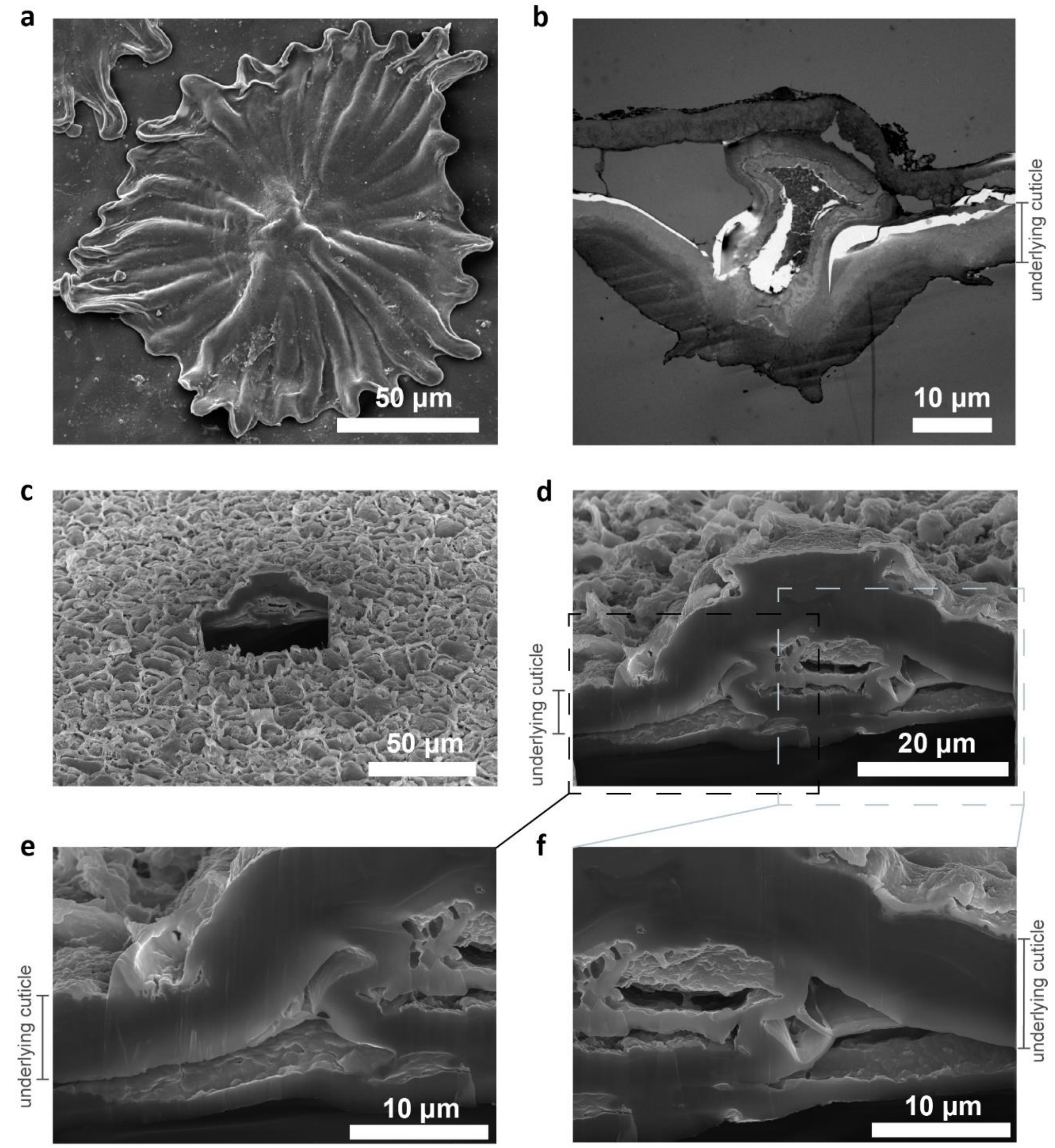

Supplementary Figure 13. Electron microscopy images of an enzymatically isolated adaxial side olive leaf cuticle focusing locally at trichomes (peltate hair). (a) Top-view SEM image of the outer cuticular side of an olive cuticle. (b) TEM image focusing at the cross-section of a trichome. (c) Overview FIB-SEM image of the inner cuticular side after local milling of the sample with a Ga ion beam and (d-f) higher magnification images focusing on the cross-section of a trichome showing the presence of a dense continuous cuticular layer underneath. The FIB-SEM images were acquired with an Everhart-Thornley detector at 15 kV and at an angle of 52°.

Supplementary Table 1. Detected IR vibration bands of CNCs

Wavenumber (cm^{-1})	Band morphology [a]	Assignment	References
3338; 3288	brd	OH stretch (intra- and inter-molecular hydrogen bonds)	[42,43]
2901	brd, wk	CH, CH_2 stretch	[42,43]
1652	brd, wk	Adsorbed H_2O, conjugated C=O	[42-44]
1429	shp, wk	CH, CH_2 bending of C_6	[42-44]
1370	shp, wk	CH bending	[42-44]
1361	shp, wk	CH bending	[44]
1336	shp	OH in plane bending	[42,44]
1316	shp	CH_2 wagging	[43,44]
1280	wk	CH bending	[42,44]
1248	wk	C=O stretching	[42,44]
1205	wk	OH in plane bending	[43,44]
1162	shp	C-O-C asymmetric stretch	[42-44]
1110	shp	Glucose ring stretch	[42-44]
1054	str, shp	C-O stretch / C-C stretch	[42-44]
1031	str, shp	C-O stretch / C-C stretch	[42-44]
984	str, brd	C-O stretch / C-C stretch	[42,44]
897	brd, wk	Glucose ring stretch	[42-44]
663	shp	OH out of plane bending	[44]

[a] Standard abbreviations (str = strong, wk = weak, brd = broad & shp = sharp) are used to describe the band morphology.

Supplementary Table 2. Detected IR vibration bands of wax-containing olive cuticles

Wavenumber (cm^{-1})	Band morphology [a]	Assignment	References
3351	brd, str	OH stretch (cutin, polysaccharides)	[32,42,43]
2918	shr, str	CH_2 stretch (cutin, waxes)	[32]
2850	shr, str	CH_2 stretch (cutin, waxes)	[32]
1731	shr, str	C=O ester stretch (cutin)	[32]
1686	shr, str	C=O stretch (triterpenoids)	[45,46]
1647	brd, str	OH bend (absorbed H_2O)	[32,45,47]
1546	shr, wk	C=C stretch (phenolics)	[32]
1462	shr, str	CH_2 bend (cutin, waxes)	[32]
1419;1315	shr, wk	CH_2 bend (cutin, waxes, polysaccharides)	[32,42,44,47]
1242	shr, str	C=O stretch (cutin, polysaccharides)	[42,44,47]
1164	shr, str	C-O-C ester stretch (cutin)	[32]
1099	shr, str	C-O-C and C-O/C-C (cutin, polysaccharides)	[32,47]
1056	shr, str	C-O-C glycosydic bond stretch (polysaccharides)	[32,42-44]
1030	shr, str	C-O/C-C stretch (polysaccharides)	[47]
998	shp, str	Ring modes (triterpenoids)	[45,46]
827	shr, wk	C-H aromatic bend (phenolics)	[32]
720	shr, wk	CH_2 bend (cutin, waxes)	[32]

[a] Standard abbreviations (str = strong, wk = weak, brd = broad & shp = sharp) are used to describe qualitatively the band morphology.

Supplementary Table 3. Radiolabeled ^3H_2O permeation data of olive cuticles using the corrected A_{ICS} to account for the effective area of the inner cuticular side

	**With Waxes**	**Without Waxes**
Permeance - 100% RH_R Inner side facing receiver [Inward] ($\times 10^{-10}$ m s^{-1})	5.15 ± 2.85	97.0 ± 12.6
Permeance - 2% RH_R Inner side facing receiver [Inward] ($\times 10^{-10}$ m s^{-1})	3.30 ± 1.46	99.8 ± 12.0
Permeance - 100% RH_R Inner side facing donor [Outward] ($\times 10^{-10}$ m s^{-1})	4.04 ± 1.40	77.9 ± 16.8
Permeance - 2% RH_R Inner side facing donor [Outward] ($\times 10^{-10}$ m s^{-1})	2.01 ± 1.04	37.4 ± 10.4
Permeability Asymmetry Factor (*PAF*) 100% RH_R [Outward/Inward] (-)	0.86 ± 0.18	0.81 ± 0.18
Permeability Asymmetry Factor (*PAF*) 2% RH_R [Outward/Inward] (-)	0.62 ± 0.13	0.37 ± 0.08

Reported values are the mean ± s.d. of n = 8 (olive with waxes) and n = 6 (olive without waxes) individual cuticular membranes measured at 25°C.

Supplementary Table 4. Radiolabeled ^3H_2O permeation data of olive cuticles without correction for the effective area of the inner cuticular side

	**With Waxes**	**Without Waxes**
Permeance - 100% RH_R<br>Inner side facing receiver [Inward]<br>($\times 10^{-10}$ m s^{-1})	5.15 ± 2.85	97.0 ± 12.6
Permeance - 2% RH_R<br>Inner side facing receiver [Inward]<br>($\times 10^{-10}$ m s^{-1})	3.30 ± 1.46	99.8 ± 12.0
Permeance - 100% RH_R<br>Inner side facing donor [Outward]<br>($\times 10^{-10}$ m s^{-1})	6.23 ± 2.16	120 ± 25.9
Permeance - 2% RH_R<br>Inner side facing donor [Outward]<br>($\times 10^{-10}$ m s^{-1})	3.10 ± 1.61	57.7 ± 16.0
Permeability Asymmetry Factor (*PAF*)<br>100% RH_R [Outward/Inward] (-)	1.33 ± 0.28	1.25 ± 0.27
Permeability Asymmetry Factor (*PAF*)<br>2% RH_R [Outward/Inward] (-)	0.95 ± 0.20	0.58 ± 0.12

Reported values are the mean ± s.d. of n = 8 (olive with waxes) and n = 6 (olive without waxes) individual cuticular membranes measured at 25 °C.

Supplementary Table 5. Radiolabeled ^3H_2O permeation data of neat SBS and SBS/CNC composite membranes containing 15 wt% CNCs

	**SBS neat**	**SBS/CNC 15 wt%**
Permeance - 100% RH_R<br>Bottom side facing receiver<br>($\times 10^{-9}$ m s^{-1})	1.17 ± 0.02	3.26 ± 0.78
Permeance - 2% RH_R<br>Bottom side facing receiver<br>($\times 10^{-9}$ m s^{-1})	1.03 ± 0.07	1.80 ± 0.29
Permeance - 100% RH_R<br>Bottom side facing donor<br>($\times 10^{-9}$ m s^{-1})	1.22 ± 0.07	3.46 ± 0.50
Permeance - 2% RH_R<br>Bottom side facing donor<br>($\times 10^{-9}$ m s^{-1})	1.10 ± 0.02	3.04 ± 0.45
Permeability Asymmetry Factor (*PAF*)<br>100% RH_R (-)	1.04 ± 0.07	1.06 ± 0.30
Permeability Asymmetry Factor (*PAF*)<br>2% RH_R (-)	1.06 ± 0.08	1.69 ± 0.37

Reported values are the mean ± s.d. of n = 3 membranes measured at 25 °C.

Supplementary Table 6. Radiolabeled ^3H_2O permeation data of ivy cuticles without correction for the effective area of the inner cuticular side

	**With Waxes**
Permeance - 100% RH_R<br>Inner side facing receiver [Inward]<br>(× 10^{-11} m s^{-1})	7.77 ± 2.19
Permeance - 2% RH_R<br>Inner side facing receiver [Inward]<br>(× 10^{-11} m s^{-1})	8.39 ± 1.44
Permeance - 100% RH_R<br>Inner side facing donor [Outward]<br>(× 10^{-11} m s^{-1})	7.26 ± 1.06
Permeance - 2% RH_R<br>Inner side facing donor [Outward]<br>(× 10^{-11} m s^{-1})	4.89 ± 1.12
Permeability Asymmetry Factor (*PAF*)<br>100% RH_R [Outward/Inward] (-)	1.01 ± 0.32
Permeability Asymmetry Factor (*PAF*)<br>2% RH_R [Outward/Inward] (-)	0.60 ± 0.16

Reported values are the mean ± s.d. of n = 10 individual cuticular membranes measured at 25 °C.

Supplementary Table 7. Dynamic Mechanical Analysis (DMA) data of olive leaf cuticles with and without waxes acquired using an amplitude of 15 μm, a frequency of 1Hz and heating rate of 3 °C min^{-1} from -100 to 150 °C

	**With Waxes**	**Without Waxes**
Storage modulus *E′* (MPa) (25 °C)	265 ± 23	89 ± 25
Storage modulus *E′* (MPa) (130 °C)	71 ± 11	32 ± 13
Sub-zero Glass transition T_g (°C)†	-7 ± 3	-8 ± 2 (shoulder peak)
Above-zero Glass transition T_g' (°C)†	N.A. (overlaid)*	24 ± 4
Melting transition T_m (°C)†	102 ± 3	N.A.

Reported values are the mean ± s.d. of n = 5 membranes. Note also that the reported values are underestimated by a factor of 2 – 3 due to overestimation of the cuticular thickness (see also Supplementary Note 2).

† Glass and melting transitions are determined by the maxima of the damping factor tanδ curves.

* Overlaid peak from the wax melting, see main text.

Supplementary References

1 Seligman, A. M., Wasserkrug, H. L. & Hanker, J. S. A new staining method (OTO) for enhancing contrast of lipid--containing membranes and droplets in osmium tetroxide--fixed tissue with osmiophilic thiocarbohydrazide(TCH). *J Cell Biol* **30**, 424-432, doi:10.1083/jcb.30.2.424 (1966).

2 Marchessault, R. H., Morehead, F. F. & Koch, M. J. Some hydrodynamic properties of neutral suspensions of cellulose crystallites as related to size and shape. *Journal of Colloid Science* **16**, 327-344, doi:10.1016/0095-8522(61)90033-2 (1961).

3 ASTM. ASTM E96 / E96M-16, Standard Test Methods for Water Vapor Transmission of Materials. (2016).

4 Greenspan, L. Humidity Fixed Points of Binary Saturated Aqueous Solutions. *Journal of Research of the National Bureau of Standards—A Physics and Chemistry* **81A**, 89-96, doi:10.6028/jres.081A.011 (1977).

5 Khanal, B. P. & Knoche, M. Mechanical properties of cuticles and their primary determinants. *Journal of Experimental Botany* **68**, 5351-5367, doi:10.1093/jxb/erx265 (2017).

6 Schreiber, L. & Schönherr, J. *Water and solute permeability of plant cuticles: measurement and data analysis*. (Springer, 2009).

7 Garvey, C. J., Parker, I. H. & Simon, G. P. On the Interpretation of X-Ray Diffraction Powder Patterns in Terms of the Nanostructure of Cellulose I Fibres. *Macromolecular Chemistry and Physics* **206**, 1568-1575, doi:10.1002/macp.200500008 (2005).

8 Segal, L., Creely, J. J., Martin, A. E. & Conrad, C. M. An Empirical Method for Estimating the Degree of Crystallinity of Native Cellulose Using the X-Ray Diffractometer. *Textile Research Journal* **29**, 786-794, doi:10.1177/004051755902901003 (1959).

9 Radhakrishnan, S. & Saini, D. R. Structure and electrical properties of polypyrrole–thermoplastic elastomer blends. *Polymer International* **34**, 111-117, doi:10.1002/pi.1994.210340115 (1994).

10 Beck, S., Méthot, M. & Bouchard, J. General procedure for determining cellulose nanocrystal sulfate half-ester content by conductometric titration. *Cellulose* **22**, 101-116, doi:10.1007/s10570-014-0513-y (2015).

11 Araki, J., Wada, M., Kuga, S. & Okano, T. Flow properties of microcrystalline cellulose suspension prepared by acid treatment of native cellulose. *Colloids and Surfaces A: Physicochemical and Engineering Aspects* **142**, 75-82, doi:10.1016/S0927-7757(98)00404-X (1998).

12 Chi, K. & Catchmark, J. M. Crystalline nanocellulose/lauric arginate complexes. *Carbohydrate Polymers* **175**, 320-329, doi:10.1016/j.carbpol.2017.08.005 (2017).

13 Sehaqui, H., Kulasinski, K., Pfenninger, N., Zimmermann, T. & Tingaut, P. Highly Carboxylated Cellulose Nanofibers via Succinic Anhydride Esterification of Wheat Fibers and Facile Mechanical Disintegration. *Biomacromolecules* **18**, 242-248, doi:10.1021/acs.biomac.6b01548 (2017).

14 Blachechen, L. S., de Mesquita, J. P., de Paula, E. L., Pereira, F. V. & Petri, D. F. S. Interplay of colloidal stability of cellulose nanocrystals and their dispersibility in cellulose acetate butyrate matrix. *Cellulose* **20**, 1329-1342, doi:10.1007/s10570-013-9881-y (2013).

15 Camarero Espinosa, S., Kuhnt, T., Foster, E. J. & Weder, C. Isolation of Thermally Stable Cellulose Nanocrystals by Phosphoric Acid Hydrolysis. *Biomacromolecules* **14**, 1223-1230, doi:10.1021/bm400219u (2013).

16 Liu, Y. *et al.* A novel approach for the preparation of nanocrystalline cellulose by using phosphotungstic acid. *Carbohydrate Polymers* **110**, 415-422, doi:10.1016/j.carbpol.2014.04.040 (2014).

17 Natterodt, J. C., Sapkota, J., Foster, E. J. & Weder, C. Polymer Nanocomposites with Cellulose Nanocrystals Featuring Adaptive Surface Groups. *Biomacromolecules* **18**, 517-525, doi:10.1021/acs.biomac.6b01639 (2017).

18 Li, B. *et al.* Cellulose nanocrystals prepared via formic acid hydrolysis followed by TEMPO-mediated oxidation. *Carbohydrate Polymers* **133**, 605-612, doi:10.1016/j.carbpol.2015.07.033 (2015).

19 Fieldson, G. T. & Barbari, T. A. The use of FTi.r.-a.t.r. spectroscopy to characterize penetrant diffusion in polymers. *Polymer* **34**, 1146-1153, doi:10.1016/0032-3861(93)90765-3 (1993).

20 Yang, J. M. & Tsai, S. C. Biocompatibility of epoxidized styrene–butadiene–styrene block copolymer membrane. *Materials Science and Engineering: C* **30**, 1151-1156, doi:10.1016/j.msec.2010.06.014 (2010).

21 Lee, W.-F. & Lee, H.-H. Surface Graft Modification of Styrene—Butadiene—Styrene Triblock Copolymer Membrane by Ultraviolet Irradiation. *Journal of Elastomers & Plastics* **42**, 49-64, doi:10.1177/0095244309349473 (2010).

22 Buonomenna, M. G. *et al.* Nanostructured Poly(styrene-b-butadiene-b-styrene) (SBS) Membranes for the Separation of Nitrogen from Natural Gas. *Advanced Functional Materials* **22**, 1759-1767, doi:10.1002/adfm.201101904 (2012).

23 Jha, A. K., Tsang, S. L., Ozcam, A. E., Offeman, R. D. & Balsara, N. P. Master curve captures the effect of domain morphology on ethanol pervaporation through block copolymer membranes. *Journal of Membrane Science* **401-402**, 125-131, doi:10.1016/j.memsci.2012.01.037 (2012).

24 Holloway, P. J. in *The plant cuticle* (eds D. F. Cutler, K. L. Alvin, & C. E. Price) 45-86 (Academic Press, 1982α).

25 Wiedemann, P. & Neinhuis, C. Biomechanics of Isolated Plant Cuticles. *Botanica Acta* **111**, 28-34, doi:10.1111/j.1438-8677.1998.tb00673.x (1998).

26 Schönherr, J. & Riederer, M. Desorption of chemicals from plant cuticles: Evidence for asymmetry. *Archives of Environmental Contamination and Toxicology* **17**, 13-19, doi:10.1007/BF01055148 (1988).

27 Bianchi, G., Vlahov, G., Anglani, C. & Murelli, C. Epicuticular wax of olive leaves. *Phytochemistry* **32**, 49-52, doi:10.1016/0031-9422(92)80104-M (1992).

28 Graniti, A. Osservazioni su Spilocaea oleagina (Cast.) Hung I. Sulla localizzazione del micelio nelle foglie di Olivo. *Phytopathologia Mediterranea* **1**, 157-165 (1962).

29 Graniti, A. Osservazioni su Spilocaea oleagina (Cast.) Hugh. III. Struttura submicroscopica della parete epidermica fogliare dell'Olivo sana e invasa dal fungo. *Phytopathologia Mediterranea* **4**, 38-47 (1965).

30 Huang, H. *et al.* Chemical Composition and Water Permeability of Fruit and Leaf Cuticles of Olea europaea L. *Journal of Agricultural and Food Chemistry* **65**, 8790-8797, doi:10.1021/acs.jafc.7b03049 (2017).

31 Riederer, M. & Schönherr, J. Accumulation and transport of (2,4-dichlorophenoxy)acetic acid in plant cuticles: I. Sorption in the cuticular membrane and its components. *Ecotoxicology and Environmental Safety* **8**, 236-247, doi:10.1016/0147-6513(84)90027-7 (1984).

32 Heredia-Guerrero, J. A. *et al.* Infrared and Raman spectroscopic features of plant cuticles: a review. *Frontiers in Plant Science* **5**, doi:10.3389/fpls.2014.00305 (2014).

33 Jeffree, C. E. in *Plant cuticles: an integrated functional approach* (ed G. Kerstiens) 33-75 (BIOS Scientific Publishers, 1996).

34 Jeffree, C. E. in *Annual Plant Reviews Volume 23: Biology of the Plant Cuticle Annual Plant Reviews* (eds M. Riederer & C. Müller) Ch. 2, 11-125 (Blackwell Publishing Ltd, 2007).

35 Fahn, A. Structural and Functional Properties of Trichomes of Xeromorphic Leaves. *Annals of Botany* **57**, 631-637, doi:10.1093/oxfordjournals.aob.a087146 (1986).

36 Palliotti, A., Bongi, G. & Rocchi, P. Peltate trichomes effects on photosynthetic gas exchange of Olea. *Plant Physiol (Life Sci. Adv.)* **13**, 35-44 (1994).

37 Karabourniotis, G., Papadopoulos, K., Papamarkou, M. & Manetas, Y. Ultraviolet-B radiation absorbing capacity of leaf hairs. *Physiologia Plantarum* **86**, 414-418, doi:10.1111/j.1399-3054.1992.tb01337.x (1992).

38 Karabourniotis, G., Kyparissis, A. & Manetas, Y. Leaf hairs of Olea europeae protect underlying tissues against ultraviolet-B radiation damage. *Environmental and Experimental Botany* **33**, 341-345, doi:10.1016/0098-8472(93)90035-E (1993).

39 Grammatikopoulos, G. & Manetas, Y. Direct absorption of water by hairy leaves of Phlomis fruticosa and its contribution to drought avoidance. *Canadian Journal of Botany* **72**, 1805-1811, doi:10.1139/b94-222 (1994).

40 Rundel, P. W. in *Physiological Plant Ecology II: Water Relations and Carbon Assimilation* (eds O. L. Lange, P. S. Nobel, C. B. Osmond, & H. Ziegler) 111-134 (Springer Berlin Heidelberg, 1982).

41 Schreiber, L. & Riederer, M. Ecophysiology of cuticular transpiration: comparative investigation of cuticular water permeability of plant species from different habitats. *Oecologia* **107**, 426-432, doi:10.1007/BF00333931 (1996).

42 Higgins, H. G., Stewart, C. M. & Harrington, K. J. Infrared spectra of cellulose and related polysaccharides. *Journal of Polymer Science* **51**, 59-84, doi:10.1002/pol.1961.1205115505 (1961).

43 Pandey, K. K. A study of chemical structure of soft and hardwood and wood polymers by FTIR spectroscopy. *Journal of Applied Polymer Science* **71**, 1969-1975, doi:10.1002/(SICI)1097-4628(19990321)71:12<1969::AID-APP6>3.0.CO;2-D (1999).

44 Liang, C. Y. & Marchessault, R. H. Infrared spectra of crystalline polysaccharides. II. Native celluloses in the region from 640 to 1700 cm.−1. *Journal of Polymer Science* **39**, 269-278, doi:10.1002/pol.1959.1203913521 (1959).

45 Ribeiro da Luz, B. Attenuated total reflectance spectroscopy of plant leaves: a tool for ecological and botanical studies. *New Phytologist* **172**, 305-318, doi:10.1111/j.1469-8137.2006.01823.x (2006).

46 Teixeira, A. C. T., Garcia, A. R., Ilharco, L. M., Gonçalves da Silva, A. M. P. S. & Fernandes, A. C. Phase behaviour of oleanolic acid/stearyl stearate binary mixtures in bulk and at the air–water interface. *Chemistry and Physics of Lipids* **160**, 45-57, doi:10.1016/j.chemphyslip.2009.04.001 (2009).

47 Szymanska-Chargot, M. & Zdunek, A. Use of FT-IR Spectra and PCA to the Bulk Characterization of Cell Wall Residues of Fruits and Vegetables Along a Fraction Process. *Food Biophysics* **8**, 29-42, doi:10.1007/s11483-012-9279-7 (2013).
