# Supplementary material for: Asymmetric water transport in dense leaf cuticles and cuticle-inspired compositionally graded membranes
Source: Nat Commun. 2021 Feb 24;12:1267. doi: 10.1038/s41467-021-21500-0 (PMC7904774; doi:10.1038/s41467-021-21500-0)
Supplement: Supplementary file 3 — Reporting Summary [file 41467_2021_21500_MOESM3_ESM.pdf]

nature research

Corresponding author(s): Prof. Dr. Christoph Weder; Prof. Dr. Lukas Schreiber

Last updated by author(s): 2021/01/26

Reporting Summary

Nature Research wishes to improve the reproducibility of the work that we publish. This form provides structure for consistency and transparency in reporting. For further information on Nature Research policies, see our Editorial Policies and the Editorial Policy Checklist.

Statistics

For all statistical analyses, confirm that the following items are present in the figure legend, table legend, main text, or Methods section.

n/a	Confirmed	
☐	☒	The exact sample size (*n*) for each experimental group/condition, given as a discrete number and unit of measurement
☐	☒	A statement on whether measurements were taken from distinct samples or whether the same sample was measured repeatedly
☐	☒	The statistical test(s) used AND whether they are one- or two-sided<br>*Only common tests should be described solely by name; describe more complex techniques in the Methods section.*
☒	☐	A description of all covariates tested
☐	☒	A description of any assumptions or corrections, such as tests of normality and adjustment for multiple comparisons
☐	☒	A full description of the statistical parameters including central tendency (e.g. means) or other basic estimates (e.g. regression coefficient) AND variation (e.g. standard deviation) or associated estimates of uncertainty (e.g. confidence intervals)
☐	☒	For null hypothesis testing, the test statistic (e.g. F, t, r) with confidence intervals, effect sizes, degrees of freedom and P value noted<br>*Give P values as exact values whenever suitable.*
☒	☐	For Bayesian analysis, information on the choice of priors and Markov chain Monte Carlo settings
☒	☐	For hierarchical and complex designs, identification of the appropriate level for tests and full reporting of outcomes
☐	☒	Estimates of effect sizes (e.g. Cohen's *d*, Pearson's *r*), indicating how they were calculated

Our web collection on statistics for biologists contains articles on many of the points above.

Software and code

Policy information about availability of computer code

Data collection	TA Universal Analysis (DMA traces), Spectrum 10 (ATR-IR spectra), LabSpec 6 software (Raman spectra), Tecnai and Imaging (TIA) software (version 4.7 SP3) (TEM imaging). SCA 20 Dataphysics software (contact angle measurements)
Data analysis	Microsoft Excel 2016, Origin 2016G, TA Universal Analysis (DMA traces), Spectrum 10 (ATR-IR spectra), LabSpec 6 software (Raman spectra), ImageJ 1.51k (size analysis of SEM, TEM images)

For manuscripts utilizing custom algorithms or software that are central to the research but not yet described in published literature, software must be made available to editors and reviewers. We strongly encourage code deposition in a community repository (e.g. GitHub). See the Nature Research guidelines for submitting code & software for further information.

Data

Policy information about availability of data

All manuscripts must include a data availability statement. This statement should provide the following information, where applicable:

- Accession codes, unique identifiers, or web links for publicly available datasets
- A list of figures that have associated raw data
- A description of any restrictions on data availability

The data are accessible through the Zenodo Sharing platform:

Field-specific reporting

Please select the one below that is the best fit for your research. If you are not sure, read the appropriate sections before making your selection.

☐ Life sciences ☐ Behavioural & social sciences ☒ Ecological, evolutionary & environmental sciences

For a reference copy of the document with all sections, see nature.com/documents/nr-reporting-summary-flat.pdf

Ecological, evolutionary & environmental sciences study design

All studies must disclose on these points even when the disclosure is negative.

Study description	Determination of the water permeation properties of non-living biological cuticular membranes isolated from Olea europaea (olive) and Hedera helix (ivy) leaf cuticles, and comparison with artificial cuticle-inspired polymer membranes.
Research sample	Artificial polymer membranes based on cellulose nanocrystals (CNCs) and poly(styrene)-block-poly(butadiene)-block-poly(styrene) (SBS)<br>Enzymatically isolated Olea europaea (olive) leaf cuticles<br>Enzymatically isolated Hedera helix (ivy) leaf cuticles<br><br>We selected olive and ivy cuticles due to their well known architecture, composition, relatively thick cuticular size, and non-porous structure.
Sampling strategy	Random sampling of membranes. No sample-size calculations, typical number of replicates for plant cuticles adapted from previous permeation studies.
Data collection	A.K., J.B., and V.V.Z.-D. conducted the experiments and analyzed the data. A.K. prepared and characterized the artificial membranes. J.B. and V.V.Z.-D. isolated the plant cuticles and performed the water permeation experiments with radiolabeled water. D.V. and A.K. carried out the electron microscopy. All experimental instruments and collection methods are described in the main text and the supplementary information of the manuscript in detail.
Timing and spatial scale	Olive cuticles were enzymatically isolated from the adaxial side of full-grown leaves harvested in an olive grove near Siena (Italy). Ivy cuticles were isolated from full-grown leaves collected outside the Institute building in Bonn (Germany).
Data exclusions	Identification and exclusion of outliers with the 1.5xIQR rule for the permeation experiments with plant cuticles due to cracks on their structure giving rise to non-diffusional transport or solute leakage from the seals.
Reproducibility	All experiments were conducted at least twice (n = 2) using different membranes. For permeation and mechanical testing experiments, more than n = 3 different samples were used (see details and number of replicates in the MS).
Randomization	Random selection of cuticular membranes (given that they qualify the "ethanol test" for macro-cracks, see experimental details MS)
Blinding	N/A (the cuticular membranes are enzymatically isolated from the plant and therefore composite non-living materials)

Did the study involve field work? ☐ Yes ☒ No

Reporting for specific materials, systems and methods

We require information from authors about some types of materials, experimental systems and methods used in many studies. Here, indicate whether each material, system or method listed is relevant to your study. If you are not sure if a list item applies to your research, read the appropriate section before selecting a response.

Materials & experimental systems

n/a	Involved in the study	
☒	☐	Antibodies
☒	☐	Eukaryotic cell lines
☒	☐	Palaeontology and archaeology
☒	☐	Animals and other organisms
☒	☐	Human research participants
☒	☐	Clinical data
☒	☐	Dual use research of concern

Methods

n/a	Involved in the study	
☒	☐	ChIP-seq
☒	☐	Flow cytometry
☒	☐	MRI-based neuroimaging
